# Gestational Diabetes Mellitus in Southeast Asia: A Scoping Review

**DOI:** 10.3390/ijerph18031272

**Published:** 2021-01-31

**Authors:** Thubasni Kunasegaran, Vinod R. M. T. Balasubramaniam, Valliammai Jayanthi Thirunavuk Arasoo, Uma Devi Palanisamy, Amutha Ramadas

**Affiliations:** Jeffrey Cheah School of Medicine and Health Sciences, Monash University Malaysia, Bandar Sunway 47500, Malaysia; Thubasni.kunasegaran@monash.edu (T.K.); vinod.balasubramaniam@monash.edu (V.R.M.T.B.); t.jayanthi@monash.edu (V.J.T.A.); umadevi.palanisamy@monash.edu (U.D.P.)

**Keywords:** gestational diabetes mellitus, Southeast Asia, screening, risk-factors, complications, management

## Abstract

A rapid increase in the prevalence of gestational diabetes mellitus (GDM) has been associated with various factors such as urbanization, lifestyle changes, adverse hyperglycemic intrauterine environment, and the resulting epigenetic changes. Despite this, the burden of GDM has not been well-assessed in Southeast Asia. We comprehensively reviewed published Southeast Asian studies to identify the current research trend in GDM in this region. Joanna Briggs Institute’s methodology was used to guide the scoping review. The synthesis of literature findings demonstrates almost comparable clinical evidence in terms of risk factors and complications, challenges presented in diagnosing GDM, and its disease management, given the similarities of the underlying population characteristics in Southeast Asia. Evidence suggests that a large proportion of GDM risk in women may be preventable by lifestyle modifications. However, the GDM burden across countries is expected to rise, given the heterogeneity in screening approaches and diagnostic criteria, mainly influenced by economic status. There is an urgent need for concerted efforts by government and nongovernmental sectors to implement national programs to prevent, manage, and monitor the disease.

## 1. Introduction

The global prevalence of gestational diabetes mellitus (GDM) is increasing at an alarming rate. According to the recent update by the International Diabetes Federation (IDF), 21.3 million live births (16.2%) were affected by hyperglycemia in pregnancy, whereby 75–90% of these pregnancies were GDM [1]. GDM is defined as “any degree of glucose intolerance with onset or first recognition during pregnancy” and remains below the cut-off value for manifest diabetes [2,3]. This complex definition of GDM includes women who have developed glucose intolerance during pregnancy or have undiagnosed pre-existing diabetes before pregnancy. Women with a GDM history have a higher risk of weight gain, preeclampsia, and cesarean sections, and half of this population will develop long-term complications, such as type 2 diabetes mellitus (T2DM) 5–10 years post-delivery.

Asia is the largest and most occupied continent (60% of the world’s population), with an increasing prevalence of GDM [4], with 30% of this population encompassing the Eastern and Southeastern subregions [5], which contribute approximately 80% to the Asian economy. The prevalence of GDM among Asian countries varies depending on the population characteristics such as maternal age, socioeconomic status, race/ethnicity, body composition, screening approaches, diagnostic benchmarks [6], presence of T2DM [7], and genetic factors, as well [8].

While the relationship between Asians and GDM has been previously described [9,10], it remains onerous to fragment evidence relating to GDM among specific Asian subregions. The term “Asian” is complex and refers to individuals from different regions, such as South Asia, Central Asia, North Asia, and Southeast Asia. Despite this, most studies group individuals of Asian origin into a single entity, regardless of region or country of birth [11]. These subgroups are diverse in terms of ethnicity, culture, and dietary backgrounds [12], making the combination of results irrelevant to a certain point. To further add to this controversy, women of the same Asian ethnicity but born in developed non-Asian countries were classified into the same groups, despite significant socioeconomic differences between these groups [13].

Such variations in attributes have led to confusion and a paucity of information about GDM in specific Asian subgroups, such as women born in Southeast Asian countries, which is the group of interest for our scoping review. The countries from this subregion have an overall higher GDM prevalence, with Thailand and Singapore showing the highest rates (24.7% and 23.5%, respectively), followed by Malaysia (22.5%) and Vietnam (21.3%), according to the IDF report in 2019 [1]. These countries are experiencing a rapid transition in socioeconomics and expansion in nutrition aspects, thus providing a reason to prioritize the current evidence of GDM in its population [1,4]. Therefore, this scoping review aims to synthesize the available data in GDM among Southeast Asian women over the past decade, explicitly targeting epidemiological, biomedical, and clinical evidence.

## 2. Methods

### 2.1. Study Design

This scoping review was conducted using the Joanna Briggs Institute methodology [14], a relatively newer approach to cluster an area of interest of the available evidence and identify knowledge gaps [15]. We began by specifying one or more aims, followed by determining the selected literature’s inclusion and exclusion criteria. The search strategies used were then identified, followed by data extraction, a discussion of findings, and a determination of the review’s limitations [16].

### 2.2. Search Strategy

The following databases were used as the sources of information for this review: Ovid EMBASE, MEDLINE, PubMed, Scopus, and Web of Science. The search was conducted using the following keywords or search terms: “gestational diabetes”, “gestational diabetes mellitus”, “Southeast Asia”, “Malaysia”, “Singapore”, “Thai”, “Thailand”, “Myanmar”, “Burma”, “Burmese”, “Cambodia”, “Laos”, “Laotian”, “Vietnamese”, “Vietnam”, “Brunei”, “Filipino”, “Philippines”, and “Indonesia”. Additionally, we included keyword combinations using Boolean operators, truncation, phrase searching, and Medical Subject Headings in the search. The search considered all relevant peer-reviewed articles written in English. The search was restricted to human samples only. A sample search done using the Ovid Medline database is provided (Table 1) and comparable strategies were used for other databases. 

### 2.3. Inclusion and Exclusion Criteria

As this review aims to elucidate the evidence in GDM, the searches were confined to articles published from 1 January 2010 to 25 May 2020. The selection of material for review was based on the following inclusion criteria: the material is relevant to GDM, such as prevalence, screening, risk factors, complications, and management; published in English-language publications; and based on original research and a peer-reviewed study. Publications not written in English or lacking focus on GDM inputs were excluded. Similarly, the review excluded materials such as editorials, conference abstracts, opinion statements, and academic theses. We also screened for references of retrieved articles, including topic-related review articles that could have been missed in the initial search.

### 2.4. Study Selection and Data Extraction

Several criteria were employed for the literature inclusion. Eligible articles met the following inclusion criteria:

- articles published in scientific journals between 1 January 2010 to 25 May 2020.
- articles published in English due to the limitation of resources for translation.
- used randomized-controlled trial, observational, cross-sectional, retrospective, or prospective study designs.
- outcome measures included prevalence, screening methods, risk factors, complications, or management of disease related to GDM.
- pharmacological or non-pharmacological interventions

As this is a qualitative review, the data extracted from the selected studies were not statistically combined and reanalyzed. Instead, the studies were broken down and summarized systematically according to the information extracted from each study, including the author name/s, year of publication, study location, the purpose of the study, participant details, research methodology, and outcome. Data were collated and summarized in the form of text, tables, and charts.

## 3. Results

### 3.1. Study Selection and Characteristics

**Figure 1:**
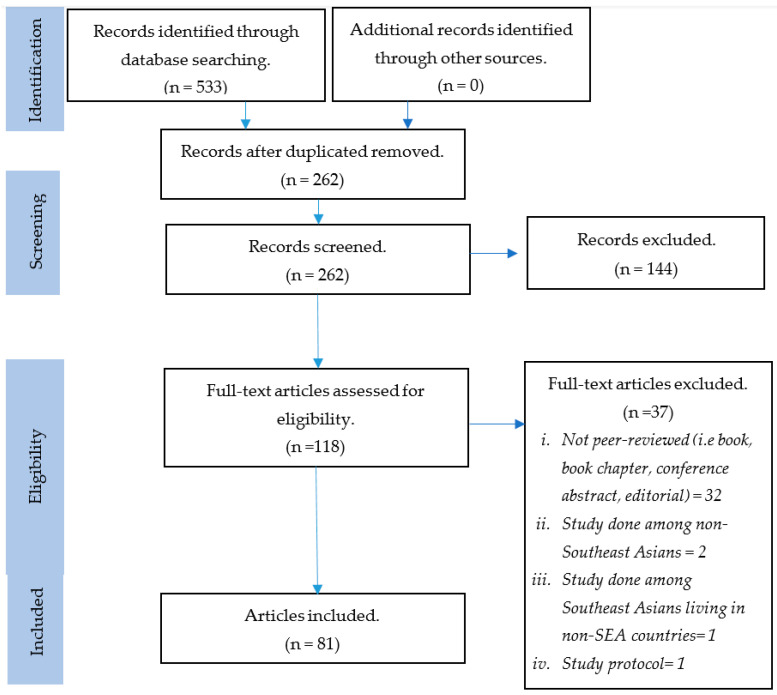
PRISMA flow chart of the article selection process.

The initial search resulted in 533 articles. We assessed 118 full-text articles for eligibility after excluding 271 duplicates and 144 articles that did not address our review topic. Thirty-seven articles were excluded at the final stage of eligibility assessment, and the remaining 81 articles were included in this review. Figure 1 shows the PRISMA flow chart of the article selection process. Supplementary Materials, Table S1 shows a full list of studies included.

**Figure 2:**
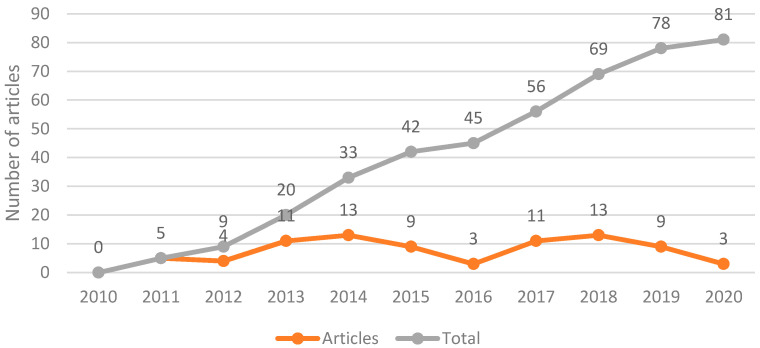
Distribution of articles by year of publication.

**Figure 3:**
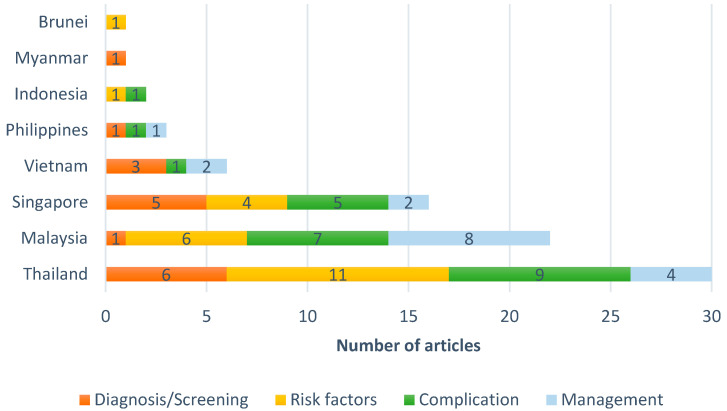
Distribution of articles by geographic location.

Figure 2 and Figure 3 display the finalized articles’ distributions according to the year of publication and geographic location, respectively. The field of GDM has recently gained attention and an expanding volume of literature, with 60% of the included studies published in the last five years. Most of the retrieved studies were conducted in Thailand (30), Malaysia (22), and Singapore (16), followed by Vietnam (6) studies. The studies primarily focused on complications (24), risk factors (23), screening/diagnosis (17), and management (17). The data in these studies were collected in hospital settings via retrospective and prospective cohort study designs.

### 3.2. Research Domains

#### 3.2.1. GDM Screening and Diagnosis

Several screening and diagnostic tests for GDM have been described over the past decades. A distinction is generally made between screening tests and diagnostic tests. A screening test can be performed on either a selective or a universal basis. Women with a positive screening test result continue with a diagnostic test, which is more extensive and demanding. Hence, the prevalence of GDM in women who underwent the diagnostic test is higher, resulting in a higher positive predictive value. In general, screening and diagnosis are performed between 24 and 28 weeks, because, at this point in gestation, a pregnancy’s diabetogenic effect manifests. There is sufficient time remaining in the pregnancy for therapy to exert its effects [17].

**Figure 4:**
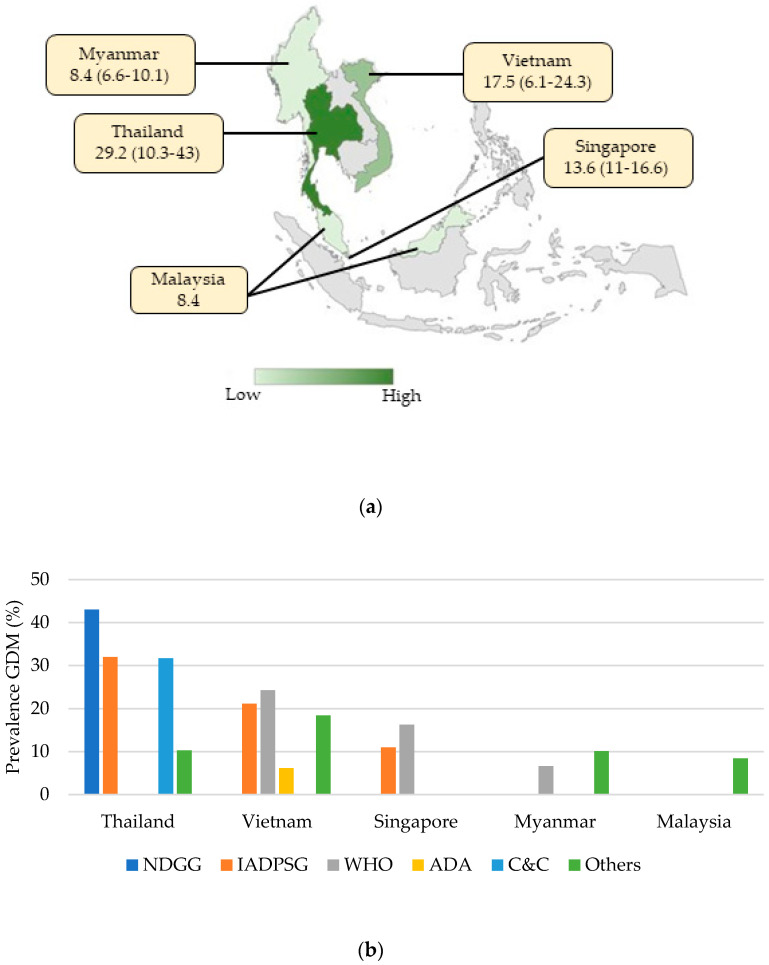
(**a**) Southeast Asian prevalence of GDM in 2010–2020. Note: Median (interquartile range) prevalence (%) of gestational diabetes mellitus (GDM) in 2010–2020. A literature search was conducted for eligible studies on the prevalence of GDM from 1 January 2010 to 25 May 2020 to capture the contemporary burden of GDM. Among the eligible studies that met the search criteria, data from countries reported in the studies were included to derive country-specific estimates for GDM prevalence. The country-specific prevalence of GDM was estimated by calculating the median prevalence of country-specific estimates within each country. (**b**) Country-specific prevalence of GDM according to different diagnostic criteria. Note: Graph of the prevalence of gestational diabetes mellitus (GDM) in selected countries according to the Carpenter–Coustan criteria (C&C), International Association of Diabetes and Pregnancy Study Groups (IADPSG) criteria, National Diabetes Data Group (NDDG) criteria, WHO 1999/2013 criteria, and International Classification of Diseases codes and local guidelines or criteria (other). A literature search was conducted for eligible studies on the prevalence of GDM from 1 January 2010 to 25 May 2020 to capture the recent burden of GDM. Among the eligible studies that met the search criteria, data from countries reported in the studies were included to derive country-specific GDM prevalence estimates based on different diagnostic criteria. The median of all available source data was used if more than one estimate of GDM prevalence was available for a country.

In our findings, Thailand and Vietnam reported the highest prevalence of GDM, with a median estimate of 29.2% and 17.3%, respectively, followed by Singapore, with a median estimate of 13.8% (range 11–11.6%). In contrast, Malaysia and Myanmar had the lowest prevalence, with a median of 8.4% (Figure 4a,b and Supplementary Materials, Table S2). Several studies in Southeast Asian countries have reported that risk-based selective screening for GDM-based screening is inappropriate. It does not precede disease complications [18,19,20]; thus, universal screening should be ideally adopted. Although universal screening may incur additional costs and resources that pose challenges for most Southeast Asian countries [21], Singapore researchers suggested that it could be more cost-effective in the long run [22].

Another screening method that was evaluated is the use of glycated hemoglobin (HbA1c) criteria. The use of HbA1c as a screening tool for GDM is still a contentious issue due to its low sensitivity and specificity. However, Poo et al. [23] concluded that a first trimester HbA1c of less than <5.2% may be useful as an additional screening tool to exclude low-risk Singaporean women from further testing of GDM in a later pregnancy period. However, women with HbA1c of 5.2% or higher would likely still need a confirmatory Oral Glucose Tolerance Test (OGTT) between 24 and 28 weeks of gestation.

Another screening method widely used by the Southeast Asian regions is using either a one-step or two-step approach. The one-step method comprises a 75- or 100-g oral glucose challenge test (75- or 100-g OGC), depending on the diagnostic guidelines. In comparison, the two-step method begins with a 50-g OGC, and those who meet or surpass the screening benchmark would undergo 100-g OGC. The one-step approach has resulted in a higher GDM prevalence and was a more appropriate method to identify more women with underlying GDM in Thailand [24]. However, their mean birthweight was significantly higher among pregnancies with GDM diagnosed by the two-step approach, leading to the conclusion that the one-step method may not be appropriate or useful in the long term.

Similarly, De Luna et al. [25] reported that Filipino women who underwent a single-step approach (75 g) followed by the International Association of the Diabetes and Pregnancy Study Groups (IADPSG) diagnostic criteria values were not significantly associated with increased adverse maternal outcomes. In contrast, Nguyen et al. [26] reported that the rates of developing adverse maternal and neonatal outcomes among Vietnamese women vary and were significantly increased through the different diagnostic criteria used. However, they were subjected to the same single-step 75-g OGC screening method. Women with GDM, according to the European Association for the Study of Diabetes (EASD), were more likely to have macrosomic infants compared to the 2010 American Diabetes Association (ADA), IADPSG/WHO, and National Institute for Health and Care Excellence (NICE) diagnostic criteria. Furthermore, babies born to mothers with GDM appeared to be large-for-gestational age by ADA criteria or by EASD criteria compared to their counterparts in the normal group.

The “gold standard” for diagnosing GDM has always been the 100-g, three-hour Oral Glucose Tolerance Test (OGTT). This test was initially used to diagnose only “ordinary” diabetes (mainly, T2DM) and validated only for that clinical entity. When the test was first introduced for pregnant women, it was hoped that it would distinguish those who were susceptible to contracting T2DM later in life to initiate early treatment. It was not meant to be used to prevent complications during pregnancy [27].

According to O’Sullivan and Mahan [28], the original diagnostic cut-off values were based on venous blood measurements. Today, blood glucose measurements are predominantly performed on plasma. However, there are two separate cut-off values that are currently in use: the “Carpenter and Coustan (C&C) cut-off threshold values” [29] and the “National Diabetes Data Group (NDDG) cut-off threshold values.” C&C cut-off threshold values are lower compared to the NDDG criteria values [27]. Two abnormal values are needed for the diagnosis of GDM [28].

In Thailand, the effectiveness of the 100-g, three-hour OGTT in the Carpenter–Coustan (C&C) and National Diabetes Data Group (NDGG) criteria in predicting pregnancy outcomes was studied by Boriboonhirunsarn et al. [30] and Luengmettakul et al. [31]. The prevalence of GDM increased by 22.2% and 32.76%, respectively, by using C&C criteria compared to NDGG criteria. However, in another study using the modified NDGG criterion, which uses only the fasting blood glucose (FBG) detection and 100-g, two-hour OGTT, it showed the highest sensitivity and accuracy among Thai pregnant women. It can also detect an equivalent percentage of maternal and fetal/neonatal complications compared to the original NDGG criterion [32]. In addition, Heetchuay et al. [33] reported that some women in Thailand experienced only a single abnormal value of 100-g OGTT and did not meet the diagnostic criteria for GDM. However, they were still at increased risk of adverse pregnancy outcomes associated with the disease.

Similarly, researchers from Vietnam have compared the IADPSG criterion, requiring one positive value on the 75-g glucose tolerance test, to the ADA criterion, requiring two positive values. GDM was diagnosed in 6.1% by the ADA criterion and 20.3% by the IADPSG criterion [34]. This IADPSG criterion with a single abnormal value on the OGTT has identified women at risk of having a preterm birth or a baby requiring treatment for neonatal hypoglycemia compared to the ADA criterion.

Another criterion that is being used in Southeast Asian countries is the WHO diagnostic criteria. It uses 75-g, two-hour OGTT. Adopting the 2013 WHO criteria without the one-hour glycemia measurement reduced the reported GDM prevalence in Singapore, especially among the Chinese and Indians [35,36]. The authors also suggested that lowering the fasting blood glucose threshold value may identify women who might benefit from treatment. However, raising the two-hour threshold value may fail to identify women at increased risk of adverse pregnancy and future metabolic outcomes within a relatively short interval of four to five years after delivery.

These reviewed studies on diagnostic criteria lead to a two-hour OGTT alone being sufficient instead of a three-hour OGTT. It may reduce the workload of physicians and the cost of screening in these low-/middle-income countries. Besides that, lowering the two-hour threshold values could provide better disease management by identifying the “borderline” women at risk of adverse pregnancy outcomes, which could lead to cost savings in the healthcare sector for the long run.

#### 3.2.2. Risk Factors

**Figure 5:**
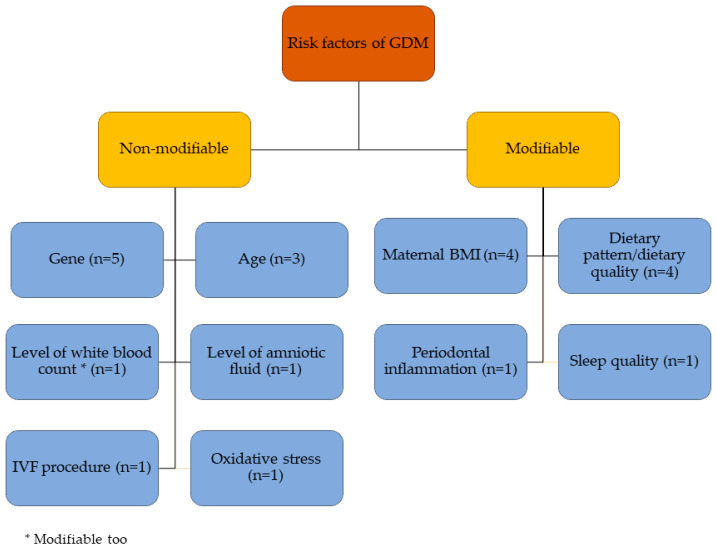
Studied risk factors of GDM in Southeast Asian countries. BMI: body mass index and IVF: in vitro fertilization.

There are many risk factors to GDM that are being accepted in other regions of the world. For example, age, body mass index (BMI), ethnic origin, diabetes mellitus type 1 (T1DM) or T2DM or GDM in a first-degree relative, and a previous history of GDM. However, the widely studied nonmodifiable risk factors among Southeast Asians are gene alteration, age, white blood count and amniotic fluid, side effects from an in vitro fertilization (IVF) procedure, and cell oxidative damages. The studied modifiable risk factors are maternal BMI, imbalanced diet, periodontal inflammation, and sleep disturbances. Figure 5 presents a summary of the reported risk factors of GDM among Southeast Asians, and the detailed list is available in Supplementary Materials, Table S3. 

Genetic alterations are strongly associated with an increased risk factor of GDM, and several single nucleotide polymorphisms (SNP) have been investigated in this regard [37]. An increased risk for GDM has been associated with a higher expression of the glucokinase regulatory gene (GCKR rs780094), modulating the enzyme responsible for regulating glucose uptake and storage [38]. Besides that, GDM patients who carried a TG/GG genotype of adiponectin SNP45 had significantly lower plasma adiponectin levels than normal patients who carried TT genotype. It denotes a possible role of the TG/GG genotype in plasma adiponectin [39]. The authors postulated that SNP45 in adiponectin could be associated with increased weight gain in antenatal patients. Subsequently, it lowers plasma adiponectin levels and increases the risk of GDM. They also suggested that adiponectin SNP45 might be varied in different ethnic groups and warrants further investigation in a multiethnic population. 

Secondly, increased inflammatory mediators and oxidative stress markers have been reported as the risk factors for GDM. The serum IGF-I level [40], maternal serum 8Isop, and TNF-α levels [41] were significantly higher in Southeast Asian GDM women, despite reasonable glycemic control. However, the serum levels of retinol-binding protein 4 (RBP4) were negatively associated with insulin resistance (IR) in pregnancies with GDM [42]. This study’s subject was from a low BMI group, and this opens the opportunity for further investigations of other ethnic groups from Southeast Asian countries with a higher BMI to detect the relationship of RBP4 levels and the degree of IR.

Thirdly, age is becoming a vital risk factor for GDM. Ages over 25 [43,44,45] or 30 [46] years old are recognized as a well-known risk factor globally, and most countries use 25 years as the threshold. However, pregnant Thai girls 16 years or younger than a group of 20 years old were reported to be at risk of developing adverse maternal outcomes, affecting their neonatal outcomes, as well [47,48,49]. One of the underlying factors is the negative socioeconomic impacts at the family and social levels leading to a low awareness of the disease and its management.

Fourthly, the rate of GDM was significantly higher among women with elevated levels of white blood cells (WBCs) in early pregnancy. However, the increased WBC is reversible, as it involves dietary factors, aside from gene influences [50]. Aside from that, Hanprasertpong et al. found that the amniotic fluid glucose (AFglu) level tends to decrease with the increasing gestational age in advanced maternal age women and could lead to the predictive risk factors for subsequent GDM [51].

The bodyweight before and during pregnancy plays a significant role in predicting GDM. Studies have shown that overweight and underweight women are more likely to have adverse pregnancy outcomes than those with average body weights. Higher maternal obesity was not only linked to GDM [52,53], but it also significantly increases gestational hypertension [52,53,54]. Besides that, high pre-pregnancy BMI without metabolic problems has been shown to increase the gestational hypertension risk. Still, it did not increase the risk of GDM or poor neonatal outcomes, since those obese women controlled their diet during pregnancy [55]. In vitro fertilization (IVF) pregnancies also have been found to independently increase the risk of GDM, especially among overweight or obese women in Singapore [56]. This finding reinforces the need to advise overweight or obese women contemplating IVF to lose excess weight before the procedure to reduce their risk of GDM and hyperglycemia-related adverse outcomes arising from there.

Poor dietary habits are also a risk factor for GDM among Southeast Asian women [57]. A higher intake level of seafood protein [58,59] and less fiber [60] in daily meals has been reported to increase the risk of GDM during pregnancy. Besides that, a combined vitamin B12 insufficiency and high folate concentration were seen among the Indian ethnic population [61]. The lower household incomes in these countries may predict the poor dietary intake quality [62] in this population.

Another risk factor is the periodontal condition linked to the increased GDM development risk among nonsmoking females [63]. It remained significant even after the additional adjustment for the family history of diabetes, pre-pregnancy body mass index, and weight gain during pregnancy. The increased inflammatory mediator levels, as pointed above, could be the underlying reason for this condition, which leads to a risk of GDM.

Lastly, psychological well-being plays a part in defining the risk factors for GDM women; for example, insufficient sleep or chronic exposure to short nocturnal sleep durations have exhibited abnormal glucose regulation among multiethnic Singaporean pregnant women [64]. Hence, it is vital to treat sleeping problems and improve sleep behaviors during pregnancy, potentially reducing the risk and burden of GDM.

#### 3.2.3. GDM-Related Complications

**Figure 6:**
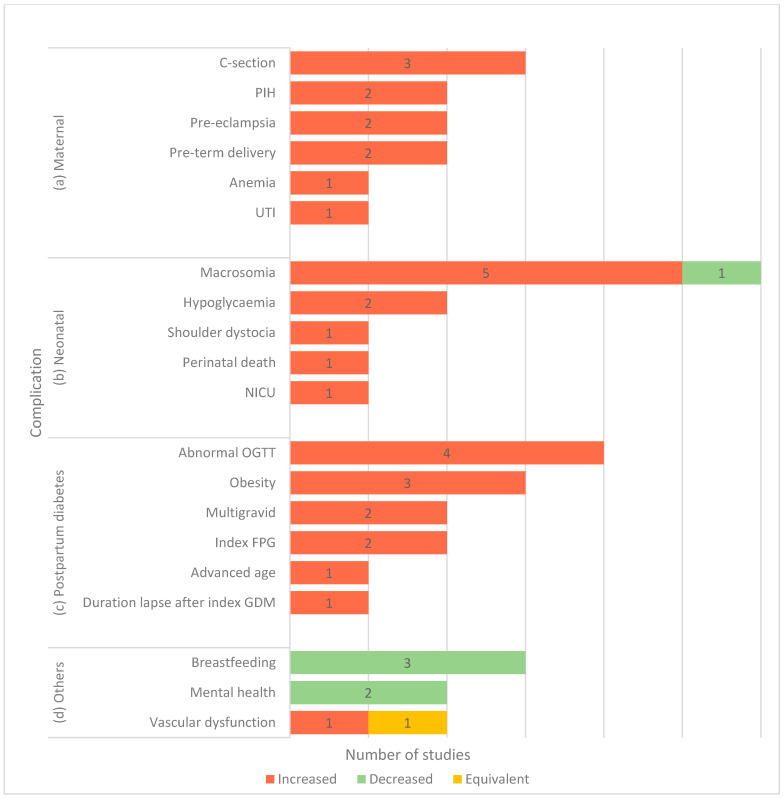
Factors associated with the complication of GDM. Note: Several influencing factors were studied under the subdomains of maternal outcomes (**a**), neonatal outcomes (**b**), postpartum diabetes (**c**), and other complications (**d**). Dec: decreased risk/incidence of complications, Inc.: increased risk/incidence of complications, and Equ: equivocal/no association. HbA1c: glycated hemoglobin, PIH: pregnancy-induced hypertension, UTI: urinary tract infection, NICU: neonatal intensive care unit, Index FPG: index fasting plasma glucose, BV dysfunction: blood vessel dysfunction, and OGTT: Oral Glucose Tolerance Test.

The purpose of screening for GDM is to diagnose and treat patients as early in a pregnancy as possible, thereby preventing complications caused by elevated blood glucose levels in pregnancy. Several pregnancy complications are thought to be caused by GDM. Most clinical studies reported complications in maternal outcomes (31%), followed by studies reporting on postpartum diabetes (28%) and complications in neonatal outcomes (26%). Other studies (15%) reported were on the complications related to impaired blood vessels, negative impacts on the breastfeeding journey, and studies on mental health deterioration in pregnant women with GDM (Figure 6 and Supplementary Materials, Table S4).

Macrosomia is one of the most mentioned problems associated with maternal GDM in Southeast Asian countries [65,66,67,68,69]. Macrosomia is an intermediate outcome, which is itself not damaging to the mother or baby. However, with macrosomia, there are chances to increase, in cesarean deliveries and instrumental deliveries, birth trauma such as brachial plexus injury or clavicular fracture neonatal hypoglycemia. Several studies reported high cesarean-section rates [65,66,70,71] in Southeast Asian countries, which affects the macrosomia incidence rates. Aris et al. [67], on the other hand, suggested that maternal weight significantly influences the risk of macrosomia incidence rather than maternal GDM. Lean pregnant women with lower gestational weight gains have less of a chance of producing macrosomia babies and a decreased risk of cesarean delivery than in women with higher gestational weight gains. In Singapore, the macrosomia occurrence was also increased in multiparous Chinese women with advanced maternal ages above 35 years and with gestational weeks of more than 40 weeks, and fathers with BMI of more than 25 [69]. It warrants physicians in other Southeast Asian regions to screen for these risk factors observed besides the maternal GDM condition. 

The increase in birth trauma in the offspring of women with GDM is thought to be caused by a higher rate of macrosomia, which predisposes pregnant women to shoulder dystocia and birth trauma. The incidence of shoulder dystocia [66] has been reported in Thailand. Similarly, babies born to a mother with a history of GDM have an increased rate of neonatal hypoglycemia [68,72], and this is the primary reason for the growing admission to the neonatal intensive care unit (NICU) [73]. Aris et al. [67] observed that an increase in maternal fasting glucose was associated with an increase in large-for-gestational age. However, there is also a need to diagnose babies with large gestational ages not due to GDM. It may assist in predicting macrosomia accurately, with its associated perinatal complications.

Perinatal mortality is also considered to be the most critical complication of GDM. Sunjaya et al. [71] reported six infant deaths among 45 GDM women. These women had significantly higher BMI before pregnancy, higher body weights before and after pregnancy, and worse glycemic profiles. These findings are similar to the above studies, which have stressed the link of maternal BMI to the poor pregnancy outcome in GDM. 

GDM also has been associated with an increase in hypertensive disorders such as preeclampsia [71,74] and diabetes-induced hypertension [66,75] compared to non-GDM women. The pathophysiology of this significantly increased risk might be linked to insulin resistance or to coexisting mutual risk factors such as obesity, advanced maternal age, and family history [76].

Another GDM-related complication that is widely observed among Southeast Asian women is postpartum diabetes. This condition was noted in high-risk women with obesity [77,78,79], multigravida [77,78], high fasting blood glucose at a diagnosis of index GDM [78,80], the presence of abnormal plasma glucose values during an OGTT [71,72,80,81], long duration lapse after index GDM [80], and being over 35 years of age [78]. Fatin et al. also reported that insulin usage, abnormal glycated hemoglobin, low postpartum follow-up visits, and neonatal intensive care unit admission were found to have significant associations with abnormal glucose tolerance at six weeks postpartum [73].

Southeast Asian women with GDM have been reported to have vascular dysfunction, as well. GDM women who were significantly older had a history of GDM, a family history of diabetes with higher pre-pregnancy BMI, lower weight gains by 26–28 weeks of gestation, and reported having small vessel dysfunction [82]. For example, abnormalities in the retinal arteriolar microvasculature in the late trimester of pregnancy. In contrast, Tengku et al. [83] found no significant association in the mean macular and retina nerve fiber layer (RNFL) thickness in GDM pregnant women compared to healthy pregnant and healthy nonpregnant women. Early onset of the disease, adequate diabetic control, and fair treatment compliance among subjects were the reasons for this scenario.

The interruption of breastfeeding has also been considered a complication among Southeast Asian women with GDM. These women have shorter breastfeeding durations than those without GDM in Thailand [84,85] and Vietnam [86]. The higher rates of obstetric and neonatal complications for both infants and GDM mothers [87], delays in the initiation of copious milk production [88], and suboptimal breastfeeding after hospital discharge [89] could be the reasons that lead to early breastfeeding cessation in this population.

Lastly, Lee et al. described that women with GDM had the highest prevalence of anxiety symptoms (39.9%), followed by depressive symptoms (12.5%) and stress symptoms (10.6%) [90], and have identified several genes modulations that lead to depression symptoms [91]. Factors such as young age, being asthmatic, and having a family history of depression and anxiety had significant associations with antenatal anxiety symptoms in women with GDM. The team also found a positive correlation between increased neonatal respiratory distress and depression symptoms in women with GDM [70]. This finding is crucial, as it gives a new dimension to the management of GDM.

#### 3.2.4. Management

**Figure 7:**
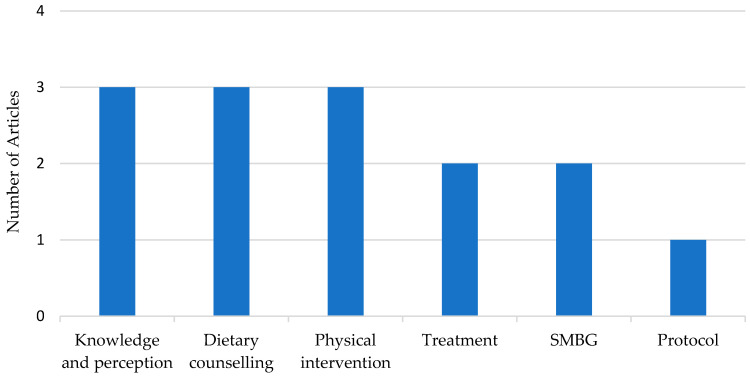
Number of articles assessing the management of GDM among Southeast Asian women. Note: SMBG; Self- monitoring blood glucose.

Figure 7 summarizes the number of studies recorded on the possible management types for GDM in Southeast Asian countries (Supplementary Materials, Table S5). Firstly, the perception and knowledge of GDM, such as those advocated by Hussain et al. [92,93] and Youngwanichsetha et al. [94], are known to play a significant role in pregnant glycemic control women. They concluded that having disease knowledge is significantly associated with better fasting blood glucose levels and education levels, substantially influencing the glycemic levels. The Hussain et al. team also found an association between a negative attitude, inadequate treatment satisfaction, and higher glycemic levels among pregnant women. Most of these patients also had difficulties in active coping measures to manage GDM [95]. Hirst et al. [96] determined that women felt confusion and anxiety for many reasons. For example, these women felt insecure, thinking the baby was at risk of death, were concerned about the transmission of GDM through breastmilk, and had an unawareness of appropriate food substitutions. Besides that, Hewage et al. pointed out that healthcare providers have less understanding of the need for compliance with the long-term maintenance of lifestyle changes [97]. This was exacerbated further by a lack of follow-up care and resources when it comes to the perceptions of responsibilities related to reducing type 2 diabetes risks among women with previous GDM.

Secondly, nutrition education and counseling on the daily dietary intake among women with a current and past history of GDM are essential to controlling the disease complications. Sangeetha et al. demonstrated that it is feasible to lower the glycemic index through nutrition education in post-GDM women [98]. These were reflected in a study by Suhaimi et al., in which they concluded that women who had a history of GDM tended to consume more low glycemic index (GI) foods than women without a history of GDM [99]. However, the delivery of nutrient recommendations regarding dietary carbohydrate intakes was inconsistent, indicating a need for consensus on dietetic practice guidelines for the management of GDM [100].

Thirdly, blood glucose self-monitoring is also a way to control glycemic levels effectively. While blood glucose self-monitoring induces anxiety among women with GDM, Southeast Asian women with GDM could overcome and tolerate this challenge [101]. Worrying about the disease impact, the elevation of glucose, and patience for the child motivated them to overcome the challenge. Besides that, a prospective study evaluated the importance of having continuous glucose monitoring (CGM) in women on insulin treatments [102]. Paramasivam et al. concluded that CGM improved the glycemic control with no significant increase in symptomatic hypoglycemia in women with more severe diseases and a higher risk of adverse maternal-fetal outcomes.

Fourthly, researchers from Thailand have differentiated the benefits of conservative versus systematic GDM management protocols based on ADA guidelines [103]. Conservative management is based on the standard guidelines for treatment by only attending to the physician’s judgment without a multidisciplinary team. In contrast, systematic management is a new protocol composed of standard guidelines for treatment with patient counseling, tightly controlling blood sugar, close monitoring, and a multidisciplinary team approach. They found that systematic management decreased hospitalizations, increased early consultation, reduced neonatal hypoglycemia prevalence, and increased postpartum diabetes mellitus (DM) surveillance by regular follow-ups.

Physical interventions have also been explored in Southeast Asia [104,105,106]. High levels of physical activities, particularly moderate-intensity activities and household/caregiving activities during pregnancy, were associated with a lower prevalence of GDM independent of sitting time [106]. Padmapriya et al. [104] associated it with lower two-hour plasma glucose levels, particularly in overweight/obese women. However, factors such as tiredness, childcare duties, and lack of time were perceived barriers to exercise in women with GDM [105]. The researchers suggested that health care professionals’ involvement in educating women with GDM on exercise can promote awareness.

Lastly, appropriate treatment options are essential for optimal glycemic control. De Luna et al. concluded that using a supplementary insulin analog was comparable to human insulin for GDM in terms of the efficacy in achieving glycemic control and can be safely used as a viable treatment option without an increased risk of hypoglycemia. However, the maternal and neonatal adverse effects were not reduced, except for the prematurity rate [107]. This finding needs further investigation on the benefits of insulin analog on improving adverse pregnancy outcomes.

Furthermore, the impact of probiotic supplements was also studied among Thai women with GDM. It lowered the fasting blood glucose level and increased the insulin sensitivity during the four-week probiotic supplements adjuvant to diet-control therapy [108]. Further exploration of probiotics’ efficacy in adverse pregnancy outcomes could open up a new treatment option in GDM.

## 4. Discussion

This scoping review was conducted to elucidate research evidence on GDM in Southeast Asian countries between the years 2010–2020. The information extracted was on the presence of risk factors with variations in biomedical, demographics, and lifestyle aspects; the diagnostic methods applied to detect GDM in the multiethnic cohort and its impact on the prevalence; the reported maternal and neonatal outcomes of women with GDM; and the challenges faced by these southeast Asian pregnant women to manage the disease efficiently.

The rising trend of GDM prevalence in Asian countries in general and the Southeast Asian region is associated with the risk factors summarized in Figure 3. Several distinctive gene modulations in the multiethnic group and unmodifiable risk factors intensify the liability of the disease. Environmental and lifestyle conditions hasten the development of disease complications. In Southeast Asia, obesity is considered a critical risk factor for chronic and noncommunicable diseases [109,110]. The above literature findings prove that the increasing trend of being overweight or obese causes adverse maternal outcomes like the risk of GDM, pregnancy-induced hypertension, increase in macrosomia, and cesarean delivery rate among Southeast Asian pregnant women. Although women with GDM are an at increased risk for T2DM, the evidence strongly suggests that T2DM and its comorbidities are preventable in this population, as the relationship between an increasing BMI and T2DM has been described in other parts of the world, even within the normal BMI category [111,112,113]. In support of that, several of the studies above concluded that the GDM risk is reduced in Southeast Asian women who engage in high physical activity levels and consume high fiber and low carbohydrate diets. Therefore, reducing being overweight or obese during pre-pregnancy should reduce diabetes-related pregnancy outcomes. Similarly, postpartum women should maintain weight loss to reduce the future risk of T2DM.

Secondly, a pregnancy in young age groups below 16 years old is considered a high-risk pregnancy and has several negative socioeconomic impacts at the family and social levels. This population is susceptible to adverse outcomes such as anemia, obstetric complications, hypertensive disorders, medical diseases, and GDM. In addition, the offspring of this group of mothers have heightened risks of metabolic syndrome. Compared with children of other ethnic origins, Southeast Asian children manifest metabolic obesity, insulin resistance, and metabolic perturbation at younger and older ages. In addition to malnutrition and lifestyle influences, the reported incidences of large-for-gestation birth weight could partially explain the reasons behind this predicament. Safe sex educational programs to reduce adolescent pregnancies and subsequent antenatal care plans to reduce the associated complications for both mother and fetus during pregnancy, delivery, and the postpartum period must be implemented for Southeast Asian reproductive-aged women.

In terms of screening and disease diagnosis challenges, the lack of consensus regarding the use of diagnostic criteria for GDM, such as NDDG [27], C&C [29], ADA [114], WHO [115], and IADPSG [116], is attributed primarily to the heterogeneity of GDM prevalence. These criteria use different screening methods, such as the one-step or two-step approach, which leads to the variations in GDM prevalence. Like the reported studies above, a recent meta-analysis of 40 studies in Europe concluded that the one-step screening method resulted in a higher prevalence of GDM than the two-step procedure [117]. Although a one-step screening type is more straightforward, less ponderous, and of lower cost, it typically overestimates the prevalence of GDM [118]. However, a two-step screening method is more voracious. It could reduce personal and societal financial burdens in the long run, despite its inconvenience for patients and increased workload for healthcare professionals [119]. Given the lack of international agreement in screening and diagnostic methods for GDM, it is exigent to develop a standardized approach to compare GDM burdens among Southeast Asian countries and, possibly, worldwide.

Furthermore, there is a need for an exceptional understanding of the benefits of precise detection and closely monitored diabetes-related complications that prevention strategies in GDM lack even among the medical community. The lack of uniform screening and diagnostic methods leads to poor maternal and offspring complications. For example, the rate of cesarean delivery and macrosomia is increasing in Southeast Asian mothers with GDM. It is crucial to manage obesity in childbearing-aged women with physical activity and dietary control to prevent adverse pregnancy outcomes. However, it is more vital to lower the two-hour OGTT threshold values to identify women at risk of adverse pregnancy events. We may reduce the low maternal and neonatal outcome incidences among women in Southeast Asia by considering this step. Whether all Southeast Asia countries can bear the additional cost of managing all identified women from this group is questionable. However, it is worthwhile to implement this step as a cost-saving method in the long run.

Mental health is also deteriorating among Southeast Asian women with GDM and is marked as a complication in GDM. In contrast, Canadian women with GDM does not significantly increase the risk of new-onset mental illness during pregnancy and postpartum [120]. However, Walmer and team [121] found Asian women with a history of GDM to have a higher risk of the onset of mental stress than other populations. It shows that ethnicity is a factor that needs consideration when it comes to the risk surveillance of mental illness. Thus, further investigation on the role of mental health in GDM development in multiethnic pregnant women is essential.

Lastly, strategic disease management with patients’ significant self-care efforts can prevent or minimize disease effects. Empowering patients and educating them about self-care and improving training courses for the medical community are also crucial for Southeast Asia countries. The challenge lies in raising the public’s awareness level on the risk factors for diabetes and then taking steps to prevent the disease. One strategy that can overcome the challenge is to utilize health promotion materials that capture different experiences of women with GDM. This method can help women, particularly at the time of diagnosis, be better prepared and health professionals to be better informed to control and manage the disease more effectively. Apart from that, systematic management, a relatively new protocol, may consume more time and create an extra workload for the physician. However, it has been proven to decrease the number of hospitalizations, increase early consultations, reduce the prevalence of neonatal hypoglycemia, and increase postpartum diabetes mellitus surveillance among women with GDM.

The proper interpretation of data is also necessary to adjust the nutrient/calorie intake, exercise intensity, or insulin doses to meet individual glycemic goals. Besides that, patients should interpret the data on their own through education provided by healthcare staff. The patients must be able to contact the medical team for data clarification if required. Thus, physicians are encouraged to continue educating women with GDM on glucose management importance and frequently monitor their glucose checking logbooks.

There are a few limitations to this review. Firstly, not all Southeast Asian countries actively participated in GDM studies from January 2010 to May 2020. Secondly, limiting the review to English language papers may have excluded papers published in other languages, thus possibly missing pertinent information. Additionally, each study reviewed had its populations, contexts, and concepts used in their methodology, in which the quality cannot be assured. Lastly, it was not feasible to conduct a thorough and comprehensive literature synthesis, given the large volume of articles identified in our review. Despite these limitations, we could retrieve literature from the past decade using broad search terms and five bibliographic databases. Most of this reviewed literature consisted of larger sample sizes that gave strength to our scoping review.

## 5. Conclusions

This scoping review highlighted the recent evidence of GDM in Southeast Asian countries. We targeted many study scopes, such as risk factors involved among these multiethnic women, the feasibility and acceptability of the current diagnosis and screening methods, complications reported within the recent years, and the findings of different approaches to managing the disease. Several recommendations for future directions are outlined from the results of this scoping review.

Firstly, it is challenging to compare the prevalence among Southeast Asia countries or regions with different diagnostic guidelines. The traditional risk factor-based GDM screening is flawed and insufficient for GDM diagnosis. Therefore, more research is needed on comparing the effects of universal versus risk factor-based screening for GDM in Southeast Asian ethnic groups on maternal and fetal outcomes. Furthermore, the long-term significance of a diagnosis of GDM using the CC/NDGG/WHO/IADPSG/ADA criteria in predicting the future risk of T2DM and other maternal and fetal outcomes among Southeast Asian women was not extensively studied. Future research, in hand with government bodies, should be initiated to examine the short- and long-term benefits, potential harms and opportunities, screening costs, and treating GDM in these low-/middle-income setting countries. It would be a deciding factor to selecting a generalized and unique diagnostic criterion to reduce the risk of pregnancy-related complications for both mother and child in Southeast Asian regions.

Secondly, further studies with more detailed information on dietary intake and body fat distribution are warranted to explore the underlying mechanisms by which Southeast Asian women have an increased risk of developing GDM. Thirdly, safe sex education programs at younger ages to reduce adolescent pregnancies and subsequent antenatal care plans to reduce the associated complications for both mother and fetus are essential and must be implemented for Southeast Asian countries.

Lastly, further studies concerning guidance for initiating personalized management treatment options are highly needed. Recommendations on healthy food choices according to the patients’ household income, the beneficial effects of probiotics, and physical activity interventions would support medical practitioners in managing the disease effectively. Communicating and ensuring a clear understanding of the disease risks and preventive methods to their patients will provide effective GDM control and reduce maternal and neonatal complications. On the other hand, women with GDM must take real responsibility in strictly controlling their glucose levels. Failure to do so will lead to a poorly controlled glycemic level and affect their offspring and, eventually, lead to a vicious cycle of diabetes.

## Figures and Tables

**Table 1:** Sample search using the Ovid Medline database.

No	Query	Results
1	Laotian.mp. or Laos	2015
2	Philippines/or filipino.mp	9174
3	Malaysia/or Malaysian mp.	15,948
4	Thai.mp	12,496
5	Thailand.mp. or Thailand/	32,699
6	Singapore/or Singaporean.mp	13,121
7	Cambodian.mp	1376
8	Cambodia.mp. or Cambodia	4234
9	Indonesia/or Indonesian.mp	11,202
10	Brunei/or Bruneian.mp.	220
11	Vietnamese.mp	4633
12	Vietnam/or Vietnam.mp	16,231
13	Burma.mp. or Myanmar	2784
14	Burmese.mp	767
15	1 or 2 or 3 or 4 or 5 or 6 or 7 or 8 or 9 or 10 or 11 or 12 or 13 or 14	105,265
16	Asia, Southeastern/or southeast Asian.mp	9951
17	Southeast Asia.mp.	7817
18	16 or 17	15,766
19	Gestational diabetes mellitus.mp or Diabetes, Gestational/	11,497
20	15 or 18	114,813
21	19 or 20	147

## Data Availability

Data sharing not applicable.

## Supplementary Materials

The following are available online at https://www.mdpi.com/1660-4601/18/3/1272/s1: Table S1: Summary of the recent evidence on GDM in Southeast Asia. Table S2: Studies reporting the diagnosis and screening for GDM in Southeast Asia (*n* = 17). Table S3: Studies reporting the risk factors for GDM in Southeast Asia (*n* = 23). Table S4: Studies reporting the complications of GDM in Southeast Asia (*n* = 24). Table S5: Studies that report the management of GDM in Southeast Asia (*n* = 17).
