# Supplementary material for: Gestational Diabetes Mellitus in Southeast Asia: A Scoping Review
Source: Int J Environ Res Public Health. 2021 Jan 31;18(3):1272. doi: 10.3390/ijerph18031272 (PMC7908368; doi:10.3390/ijerph18031272)
Supplement: Supplementary file 1 [file ijerph-18-01272-s001.pdf]

Table S1: Summary of recent evidence on GDM in Southeast Asia

Aspect	Author	Year	Country
Diagnosis	Hansarikit et al [32]	2011	Thailand
	Hirst et al [34]	2012	Vietnam
	Arora et al [18]	2013	Thailand
	Tran et al [21]	2013	Vietnam
	Boriboonhirunsarn et al [30]	2014	Thailand
	Chen et al [22]	2014	Singapore
	Chong et al [10]	2014	Singapore
	Gilder et al [19]	2014	Myanmar
	Yew et al [36]	2014	Singapore
	Luengmettakul et al [31]	2015	Thailand
	De Luna et al [25]	2017	Philippine
	Ganeshan et al [20]	2017	Malaysia
	Heetchuay et al [33]	2017	Thailand
	Chi et al [35]	2018	Singapore
	Luewan et al [24]	2018	Thailand
	Nguyen et al [26]	2019	Vietnam
	Poo et al [23]	2019	Singapore
Risk factors	Adzura et al [40]	2011	Malaysia
	Kongubol et al [54]	2011	Thailand
	Low et al [39]	2011	Malaysia
	Khovidhunkit et al [42]	2012	Thailand
	Chantrapanichkul et al [47]	2013	Thailand
	Chokwiriyachit et al [63]	2013	Thailand
	Htwe et al [52]	2013	Brunei
	Ismail et al [37]	2013	Malaysia
	Lim et al [53]	2013	Thailand
	Pattanathaiyanon et al [50]	2014	Thailand
	Traisrisilp et al [48]	2015	Thailand
	de Seymour et al [58]	2016	Singapore
	Hanprasertpong et al [51]	2016	Thailand
	Amiruddin et al [60]	2017	Indonesia
	Cai et al [56]	2017	Malaysia
	Cai et al [64]	2017	Singapore
	Pang et al [59]	2017	Singapore
	Chawanpaiboon et al [49]	2018	Thailand
	Jamalpour et al [38]	2018	Malaysia
	Lai et al [61]	2018	Singapore
	Rueangdetnarong et al [41]	2018	Thailand
	Somprasit et al [55]	2015	Thailand
	Yong et al [57]	2020	Malaysia
Complication	Sia et al [76]	2011	Philippine
	Chew et al [80]	2012	Malaysia
	Youngwanichsetha et al [72]	2014	Thailand
	Youngwanichsetha et al [84]	2013	Thailand
	Youngwanichsetha et al [78]	2013	Thailand
	Aris et al [68]	2014	Singapore
	Asvanarunat et al [65]	2014	Thailand
	Yadav et al [69]	2014	Malaysia

	Aris et al [67]	2015	Singapore
	Hanprasertpong et al [74]	2015	Thailand
	Srichumchit et al [66]	2015	Thailand
	Ruksasakul et al [79]	2016	Thailand
	Li et al [82]	2017	Singapore
	Wanthong et al [81]	2017	Thailand
	Li et al [75]	2018	Singapore
	Sunjaya et al [71]	2018	Indonesia
	Fatin et al [73]	2019	Malaysia
	Jirakittidul et al [85]	2019	Thailand
	Lee et al [90]	2019	Malaysia
	Lee et al [91]	2019	Malaysia
	Nguyen et al [86]	2018	Vietnam
	Lee et al [70]	2020	Malaysia
	Tengku et al [83]	2020	Malaysia
Management	Hirst et al [96]	2012	Vietnam
	Sangeetha et al [98]	2013	Malaysia
	Farhanah et al [100]	2014	Malaysia
	Hussain et al [95]	2014	Malaysia
	Youngwanichsetha et al [94]	2014	Thailand
	Hussain et al [92]	2015	Malaysia
	Hussain et al [93]	2015	Malaysia
	Panpitpat et al [103]	2015	Thailand
	Padmapriya et al [104]	2017	Singapore
	Youngwanichsetha et al [101]	2017	Thailand
	Arasoo et al [105]	2018	Malaysia
	De Luna [107]	2018	Philippine
	Hewage et al [97]	2018	Singapore
	Nguyen et al [106]	2018	Vietnam
	Paramasivam et al [102]	2018	Malaysia
	Kijmanawat et al [108]	2019	Thailand
	Shuhaimi et al [99]	2019	Malaysia

Table S2: Studies reporting diagnosis and screening for GDM in Southeast Asia (n=17)

Author	Study aim	Study design / Setting / Sample size	Main findings
Hansarikit et al [32]	To study the sensitivity and specificity of the modified 100-g oral glucose tolerance test for diagnosis of gestational diabetes mellitus (GDM).	• Retrospective cohort<br>• Hospital<br>• N=511; n=308 (GDM) & n=203 (control)	• Modified II criterion (fasting glucose and 2-hour glucose) has the highest sensitivity of 96.8%, and the highest accuracy of 90.8%.<br>• The modified II criterion can detect the same proportion of maternal and neonatal complications, compared to the National Diabetes Data Group (NDDG) criterion.
Hirst et al [34]	To determine prevalence and outcomes in urban Viet Nam by comparing International Association of the Diabetes and Pregnancy Study Groups (IADPSG) criterion, requiring one positive value on the 75-g glucose tolerance test, to the 2010 American Diabetes Association (ADA) criterion, requiring two positive values.	• Prospective cohort<br>• Hospital<br>• N=2772; n=714 (GDM) & n=2058 (control)	• GDM was diagnosed in 164 participants (6.1%) by the ADA criterion, 550 (20.3%) by the IADPSG criterion.<br>• Women with GDM and borderline (GDM by the IADPSG criterion but not the ADA criterion) were more likely to deliver preterm (OR=1.49, 95% CI=1.16–1.91) and (OR= 1.52, 95% CI=1.03–2.24), respectively.<br>• Women with GDM and borderline GDM more likely to have clinical neonatal hypoglycemia (OR=4.94, 95% CI= 3.41–7.14) and (OR=3.34, 95% CI= 1.41–7.89), respectively.<br>• For large for gestational age, the ORs were 1.16 (0.93–1.45) and 1.31 (0.96–1.79) for both groups of women with GDM and borderline GDM.<br>• There was no significant difference in large for gestational age, death, severe birth trauma, or maternal morbidity between the groups. Women with GDM underwent more labour inductions (OR=1.51, 95% CI=1.08–2.11).
Arora et al [18]	To estimate the prevalence of GDM by using universal	• Cross-sectional study<br>• Hospital	• Among GDM cases, 21.8% had no risk factor.

	screening and to show the diagnostic value of the clinical risk factors.	• N=593; n=714 (GDM) & n=2058 (control)	• Having one risk factor double the chance of having GDM, while having three risk factors gives 42.9% chance of having GDM.<br>• Having at least one risk factor could allow better detection with sensitivity of 78.2, specificity of 49.8 that would produce 52.8% of pregnant women at risk.<br>•
Tran et al [21]	To compare the discriminative power of prognostic models for early prediction of women at risk for the development of GDM using four currently recommended diagnostic criteria based on the 75-g OGTT and to described the potential effect of application of the models into clinical practice.	• Cross-sectional study<br>• Hospital<br>• N=2772; n=1980 (GDM) & n=792 (control)	• There were 164 women diagnosed with GDM by the ADA criteria, (OR=5.9, 95% CI= 5.0–6.8); 565 by the IADPSG criteria (OR= 20.4, 95% CI=18.9–21.9); 577 by the ADIPS criteria (OR 20.8, 95% CI=19.3–22.3); and 674 by the WHO criteria (OR=24.3, 95% CI=22.7– 25.9)The ADA prognostic model, consisting of age and BMI at booking had the best discriminative power (area under the curve of 0.71) and the most favorable cost-effective ratio if implemented in clinical practice.<br>• Selective screening of women for GDM using the ADA model with a risk threshold of 3% gave 93% sensitivity for identification of women with GDM with a 27% reduction in the number of OGTTs required.
Boriboonhirunsarn et al [30]	To evaluate and compare the increase in the prevalence of GDM and the pregnancy outcome using CC criteria and NDDG criteria.	• Retrospective study<br>• Hospital<br>• N=640; n=145 (GDM) & n=495 (control)	• Prevalence of GDM increased by 22.2% using CC criteria.<br>• The change was 27.6% at the initial test and 31.5% at repeat tests during 24–28 weeks of gestation.<br>• Infant birth weight in GDM diagnosed by either NDDG or CC criteria was significantly higher than in the negative OGTT group ($p < 0.001$).<br>• Rates of macrosomia were comparable.<br>• Neonatal hypoglycemia was 14.6% in the NDDG group, 8.2% in CC only group, and 4.6% in negative OGTT group ($p < 0.001$).
Chen et al [22]	To compare the different types of screening and its cost effectiveness for GDM.	• Retrospective cohort<br>• Cohort data<br>• N= 924 (GDM)	• Relative to targeted screening using risk factors, universal screening generates an incremental cost-effectiveness ratio (ICER) of $USD10 630/QALY gained.<br>• Disease prevalence rates and intervention effectiveness of glycemic management have the biggest impacts on the ICERs.

Chong et al [10]	To compare the proportion of GDM using universal versus selective (high-risk for GDM) screening among Asian ethnic group.	• Retrospective study<br>• Hospital<br>• N=1136; n=326 (GDM) & n=810 (control)	• Universal screening detected significantly more cases than high-risk screening (OR= 2.2, 95% CI= 1.7-2.8), particularly for Chinese women (OR = 3.5,95% CI= 2.5-5.0).<br>• Pre-pregnancy BMI > 30 kg/m2 (adjusted OR = 3.4, 95% CI= 1.5-7.9) and previous GDM history (adjusted OR = 6.6, 95% CI=1.2-37.3) were associated with increased risk of GDM in Malay women while GDM history was the only significant risk factor for GDM in Chinese women (adjusted OR = 4.7, 95% CI= 2.0-11.0).
Gilder et al [19]	To determine the prevalence of GDM in refugee camp setting.	• Prospective study<br>• Refugee camp<br>• N=221; n=23 (GDM) & n=205 (control)	• Prevalence of GDM was (OR=10.1, 95% CI= 6.2-14.0] when the cut-off determined by the HAPO trial was applied.<br>• Applying the older WHO criteria yielded a prevalence of (OR=6.6, 95% CI= 3.3-9.8).
Yew et al [36]	To determine the impact of the new 2013 World Health Organization (WHO) criteria for diagnosis of GDM on prevalence and pregnancy outcomes of GDM in Asian ethnic groups, compared to the 1999 WHO criteria.	• Retrospective cohort study<br>• Hospital<br>• N=855; n=536 (GDM) & n=319 (control)	• The prevalence of GDM was reduced from 28.8% to 21.1% when the 2013 criteria were used. 10.2% women were reclassified from GDM to normal using the 2013 criteria, and 2.6% were reclassified from normal to GDM, giving a net reclassification rate of 12.8%.<br>• Reclassification from GDM to normal was greatest among Chinese, followed by Asian Indians, but prevalence was unchanged among Malays.<br>• Babies of mothers who were reclassified from normal to GDM were more likely to have birth weight >95th centile and shoulder dystocia.
Luengmettakul et al [31]	To compare the pregnancy outcome of patients diagnosed with GDM using the Carpenter–Coustan (CC) and National Diabetes Data Group (NDGG) criteria	• Retrospective cohort<br>• Hospital<br>• N=832; n=487 (GDM) & n=345 (control)<br>• Group 1: pregnant women with normal OGTT on both CC and National	• Incidence of GDM increased 32.76% using the CC criteria.<br>• Women in group 2 had a higher risk of neonatal hypoglycemia (OR 12.3, 95% CI 2.9, 52.73, p<0.0001), neonatal hyperbilirubinemia (OR 1.9, 95% CI 1.13, 3.08, p<0.013) compared with group 1.<br>• Pregnant women in group 3 had a lower incidence of immediate post-partum hemorrhage from vaginal delivery than group 2 (n=0%), as well as lower rates of neonatal hypoglycemia (OR 0.2, 95% CI 0.05,

		Diabetes Data Group (NDDG) criteria.<br>• Group 2: pregnant women with normal OGTT on NDDG criteria but GDM on CC criteria.<br>• Group 3: pregnant women with GDM on CC criteria after December 2012 and enrolled in glycemic control program.	0.84, p<0.05), and neonatal hyperbilirubinemia (OR 0.3, 95% CI 0.11, 0.65, p<0.05).
De Luna et al [25]	To determine the association of the threshold values set up by the IADPSG to diagnose GDM with adverse pregnancy outcomes among a cohort of Filipino women.	• Retrospective cohort<br>• Hospital<br>• N=120 GDM	• Each of IADPSG-defined cut-off values was not significantly associated with increased likelihood of having adverse maternal outcomes namely: hypertensive disorders of pregnancy (OR=0.79, 95% CI= 0.23-2.74, p<0.71), miscarriage (-), primary cesarean section (OR=0.83, 95% CI=0.39-1.73, p<0.61), operative vaginal delivery (OR=0.51, 95% CI=0.04-5.77, p<0.59), and maternal death (-).<br>• The likelihood of perinatal outcomes namely: macrosomia (OR=1.15, 95% CI=0.41-3.22, p<0.79), perinatal death (OR=0.51, 95% CI=0.04-5.77, p<0.59), prematurity (OR=0.75, 95% CI=0.24-2.32, p<0,62), birth injuries (-), congenital anomalies (OR=0.75, 95% CI=0.24-2.32, p<0.66), neonatal hypoglycemia (OR=,4 95% CI=0.43-37.26, p<0.22) jaundice (OR=1.20 95% CI=0.56-2.58, p<0.22), low APGAR score, acute respiratory distress (-) syndrome, and infection (OR=1.02, 95% CI=0.37-2.79, p<0.97) were not significantly higher even if these cut-off values were met.

Ganeshan et al [20]	To evaluate the effectiveness of the current practice of selective risk-based screening in detection of GDM.	• Cross-sectional retrospective cohort<br>• Cohort data<br>• N=22,044 (GDM)<br>•	• Maternal age of >/=25, booking bMI >/=27kg/m2, booking weight >/=80kg and previous hypertension are non-significant risk of developing GDM in Malaysia.
Heetchuay et al [33]	To determine the adverse outcomes of pregnancy in women with single abnormal value of the 100g OGTT compared with women with normal value of 100g OGTT.	• Retrospective cohort<br>• Hospital<br>• N=1185; n=395 (GDM) & n=790 (control)	• Women with single abnormal value 100g OGTT were older (31.82±4.92 vs. 30.79±5.58; p-value 0.001), had higher rate of family history of type 2 DM (36.5% vs. 30.8%; p-value 0.049) and lower mean gestational age at birth (38.07±1.55 vs. 38.32±1.38; p-value 0.005) than the control group.<br>• Adverse outcomes of pregnancy significantly occurred in the study group included higher rate of macrosomia (4.3% vs. 1.5%; p-value 0.003) and large for gestational age (LGA) (11.9% vs. 6.7%; p-value 0.002) when compared with the control group.
Chi et al [35]	To compare later maternal metabolic outcome based on different GDM diagnostic criteria.	• Retrospective cohort<br>• Hospital<br>• N=1092; n=284 (GDM) & n=808 (control)	• Compared to women without GDM by both criteria, cases reclassified to GDM by the 2013 criteria had an increased risk of neonatal jaundice requiring phototherapy (RR 2.78, 95% CI 1.32, 5.86); despite receiving treatment for GDM.<br>• Cases reclassified to non-GDM by the 2013 criteria had higher risks of prematurity (RR 2.17, 95% CI 1.12, 4.24), neonatal hypoglycemia (RR 3.42, 95% CI 1.04, 11.29), jaundice requiring phototherapy (RR 1.71, 95% CI 1.04, 2.82), and a higher rate of abnormal glucose tolerance at 4-5 years post-delivery (RR 3.39, 95% CI 2.30, 5.00).
Luewan et al [24]	To compare the prevalence and pregnancy outcomes of GDM between those screened by the "one-step" (75 gm GTT) and "two-step" (100 gm GTT) methods.	• Prospective cohort<br>• Hospital<br>• N=648 (GDM)	• Prevalence of GDM was significantly higher in the one-step group; 32.0% versus 10.3%.<br>• Baseline characteristics and pregnancy outcomes in both groups were comparable.<br>• Mean birthweight was significantly higher among pregnancies with GDM diagnosed by the two-step approach (3204 ± 555 versus 3009 ± 666 g; p <0.022).

			• The rate of large-for-date tended to be higher in the two-step group but was not significant.
Nguyen et al [26]	To estimate the prevalence of GDM and pregnancy outcomes among Vietnamese women.	• Prospective cohort<br>• Hospital<br>• N=2475; n=1131 (GDM) & n=1344 (control)	• The prevalence of GDM varied considerably by the diagnostic criteria: 6.4% (ADA), 7.9% (EASD), 22.8% (IADPSG/WHO), and 24.2% (NICE).<br>• Women with GDM according to EASD were more likely to have macrosomic infants (OR=4.35, 95% CI=1.49–12.72), despite no apparent increase in risk under other criteria.<br>• Babies born to mothers with GDM appeared to be large-for-gestational age (LGA) by ADA criteria (OR= 2.10, 95% CI=1.10–4.02) or EASD criteria (OR= 2.15, 95% CI=1.16–3.98), when compared to their counterparts in the normal group.<br>• No significant differences in maternal and other neonatal outcomes were found between the normal and GDM groups.
Poo et al [23]	To examine the use of early HbA1c in screening for GDM and adverse pregnancy outcomes	• Prospective cohort<br>• Hospital<br>• N=151; n=17 (GDM) & n=134 (control)	• HbA1c level of 5.2% (33 mmol/mol), had an 82% sensitivity, 72% specificity, 97% negative predictive value and 27% positive predictive value to predict GDM.<br>• Women with HbA1c of 5.2% (33 mmol/mol) or over 5.2% (33 mmol/mol) were older, had higher BMI and were less likely to be Chinese than those with HbA1c less than 5.2% (33 mmol/mol).<br>• There was no difference in pregnancy outcomes.

Table S3: Studies reporting the risk factors for GDM in Southeast Asia (n=23)

Author	Study aim	Study design / Setting / Sample size	Main findings
Adzura et al [40]	To study the association of serum insulin-like growth factor-I (IGF-I) with diabetic retinopathy.	• Prospective cohort<br>• Hospital<br>• N=50; n=25 (GDM) & n=25 (control)	• The serum IGF-I level was significantly elevated in GDM pregnant women compared to control pregnant women at 24 weeks (p=0.0001), at 32 weeks (p=0.007), and at 36 weeks of gestation (p=0.003).
Kongubol et al[54]	To assess whether Thai women classified as obese according to WHO's recommended body mass index (BMI) for Asians were at risk for developing gestational diabetes mellitus (GDM) and other complications.	• Prospective cohort<br>• Hospital<br>• N=240; n=69 (GDM) & n=171 (control)	• Obese Thai women were not at risk for GDM (RR=0.9, 95% CI=0.5-1.4).<br>• Relative risk of preeclampsia (RR=0.7, 95% CI=0.2-3.3) and fetal macrosomia (RR=1.4, 95% CI=0.5-4.3) in obese women were not significant.<br>• Relative risk of gestational hypertension in obese women was significant (RR=12, 95% CI=1.6 -90.8).<br>• Pre-pregnancy obesity without metabolic problems did not increase the risk for GDM, preeclampsia and fetal macrosomia in Thai women, but increases the risk to gestational hypertension.
Low et al [439]	To identify the association of SNP45TG with GDM.	• Cross-sectional retrospective cohort<br>• Hospital<br>• N=79; n=26 (GDM) & n=53 (control)	• Significant association was found between SNP45TG with GDM (x^2 = 4.038; p=0.044).<br>• Normal patients with TT genotype have significantly higher plasma adiponectin level compared to GDM<br>• G allele in SNP45 of adiponectin gene is significantly more frequent in gestational diabetic patients than normal patients (x^2 = 4.324; p = 0.038).
Khovidhunkit et al [40]	To examined whether serum levels of retinol-binding protein 4 (RBP4) were associated with IR in pregnancy.	• Prospective study<br>• Hospital<br>• N=533; n=172 (GDM) & n=361 (control)	• RBP4 levels and found that the levels were not significantly different between the GDM and non-GDM group (p>0.05).<br>• Significant positive correlations were found between RBP4 levels and gestational age at the time of OGTT (R=0.093, <=0.032), fasting triglyceride levels (R = 0.305, p< 0.001), and the amount of weight gain in pregnancy (R = 0.154, p < 0.001)<br>• Significant negative correlation was also found between RBP4 levels and high-density lipoprotein cholesterol levels (R = −0.121, p = 0.005).

Chantrapanichkul et al [47]	To assess pregnancy outcomes among adolescent girls 16 years old or younger and their newborns.	• Prospective cohort<br>• Hospital<br>• N=2161	• Anemia (OR=1.86, 95% CI = 1.52–2.26), heart disease (OR= 0.38, 95% CI 0.15–0.90), thyroid disorder (OR=0.054, 95% CI=0.01–0.40), pulmonary disease (OR= 0.89, 95% CI= 0.41–1.93), medical and obstetrics complications including gestational diabetes mellitus (OR= 0.04, 95% CI=0.01–0.29), placenta previa (OR=1.04, 95% CI = 0.06–16.60), preterm labor (OR=1.98, 95% CI, 1.55–2.53), as well as mean neonatal weight (2830.77±81.31 g and 3038.53±482.23 g; p=0.001) were statistically different between age group 16 and younger compared to the older age group.
Chokwiriyachit et al [63]	To examine the association between periodontitis and GDM among non-smoking pregnant females.	• Case-control<br>• Hospital<br>• N=100; n=50 (GDM) & n=50 (control)	• Periodontitis was significantly associated with GDM (OR=3.00, 95% CI=1.19-7.56, p<0.05).
Htwe et al [52]	To assess the impact of body mass index (BMI) on pregnancy outcomes among primigravid women.	• Prospective cohort<br>• Hospital<br>• N=1290 (GDM)	• Controlled for maternal age and smoking showed higher risk for gestational hypertension (RR=2.6, 95% CI=1.2-5.4) in the normal and high BMI groups (RR=3.7, 95% CI=1.8–7.5), and GDM among the high BMI group (RR=2.6, 95% CI=1.16.1), p<0.001).
Ismail et al [37]	To investigate the single nucleotide polymorphism (SNP) for identifying candidate genes involve in risk factors and complications of GDM.	• Retrospective cohort<br>• Hospital<br>• N=288; n=174 (GDM) & n=114 (control)	• The SNPs identified were rs10946398 (CDKAL1) in GDM risk factor with family history of DM (OR=0.49, 95% CI=0.25-0.96, p=0.006); rs328 (LPL) (OR=0.2.25, 95% CI=1.07-4.73, p=0.002); and rs1042778 (OXTR) (OR=2.62, 95% CI=1.28-5.36, p=0.022) in complications of caesarean section; rs5404 (SLC2A2) (OR=6, 95% CI=1.24-28.99, p=0.028); rs5400 (SLC2A2) (OR=4.48, 95% CI=1.01-23.10, p=0.033); and rs13306465 (IRS1) (OR=5.68, 95% CI=1.18-27.36, p=0.031) for neonatal intensive care admission.<br>• SNPs rs12255372, rs7901695 and rs7903146 from TCF7L2 gene had six times higher risk (OR, 6.40-6.53) for T2DM at postpartum.
Lim et al [53]	To evaluate potential differences in the adiposity-	• Cross-sectional study<br>• Hospital	• Higher maternal BMI was associated with elevated peripheral SBP (β =1.19, 95% CI=1.03-1.36, p=<0.001); peripheral DBP (β =0.76, 95% CI=0.63-0.89,

	blood pressure relation between ethnic groups and interaction with gestational diabetes.	• N=799; n=136 (GDM) & n=602 (control)	p<0.001); central systolic pressure (β =1.02, 95% CI=0.87-1.17, p<0.001); and central pulse pressure (β =0.26, 95% CI=0.16-0.37, p<0.001).<br>• The associations were generally stronger in Chinese women (β=1.51, 95% CI=0.16-2.87, p=0.03) for central pulse pressure) and individuals with gestational diabetes (p=0.03 for DBP and p=0.046) for central systolic pressure).
Pattanathaiyanon et al [50]	To examine the rates and risk of GDM in relation to white blood cell (WBC) count in early pregnancy.	• Retrospective cohort<br>• Hospital<br>• N=1145; n=105 (GDM) & n=1040 (control)	• The rate of GDM was significantly higher in increased WBC group (OR=2.20, 95% CI=1.39-3.47, p<0.001) compared to normal WBC group.
Traisrisilp et al [48]	To determine pregnancy outcomes among early adolescent women (aged≤ 15years) compared with those in late adolescence and adults.	• Retrospective cohort<br>• Hospital<br>• N=33777	• The rate of pregnancies complicated by diabetes mellitus was significantly higher in age group above 15 years (p<0.0001).<br>• Early adolescent pregnancy was associated with higher risks of preterm birth (RR=1.406, 95% CI=1.181–1.675, p<0.001) and (RR=2.193, 95% CI=1.853–2.594, p<0.001), fetal growth restriction (RR=1.530, 95% CI=1.103–2.123, p=0.05) and (RR=1.647, 95% CI=1.203–2.254, p=0.05) and low birthweight (RR=1.404, 95% CI=1.164–1.693, p<0.001) and (RR=1.960, 95% CI=1.637–2.348, p<0.001) when compared to those in late adolescent and adult pregnancies.
de Seymour et al [58]	To examine the cross-sectional relationship between maternal dietary patterns during pregnancy and the risk of GDM in a multi-ethnic Asian cohort.	• Prospective cohort<br>• Hospital<br>• N=909; n=160 (GDM) & n=749 (control)	• Seafood-noodle-based-diet was associated with a lower likelihood of GDM (OR=0.74, 95% CI=0,59-0.93, p<0.01)
Hanprasertpong et al [51]	(i) To evaluate the possibility of predicting subsequent GDM using Amniotic fluid glucose (AFglu);<br>(ii) To estimate AFglu cut-off levels for identifying	• Prospective cohort<br>• Hospital<br>• N=438; n=58 (GDM) & n=380 (control)	• GDM women has increased AFglu for each 1 mg/dl (OR=1.07, 95% CI 1.04-1.10, p<0.001).<br>• Risk of subsequent GDM was also increased in women aged over 36 years and in 17-18 weeks (OR=2.10, 95% CI=1.04-4.23, p=0.029) compared to 16 weeks of gestation.

	pregnancies at high or low risk for subsequent GDM in advanced maternal age.		• Depending on gestational and maternal age, AFglu levels above 51 to 75 mg/dl were at elevated risk of subsequent GDM (likelihood ratio 2.38)
Amiruddin et al [60]	To investigate the risk of fiber, coffee consumption and cigarette smoke exposure on the incidence of the prediabetes and GDM	• Retrospective cohort<br>• Hospital<br>• N=135; n=45 (GDM) & n=90 (control)	• The prediabetes and GDM risk increase by the existence of less fiber consumption history (OR=2.355, 95% CI=1.12-4.94, p=0.022) and less coffee consumption history (OR = 2.406, 95% CI= 1.10-5.25, p=0.025).<br>• No difference of the risk of the giving birth women to undergo the prediabetes and GDM between the giving birth women who have the high smoking exposure history and the low smoking exposure history (OR = 1.902, 95% CI: 0.81-4.47, p=0.137).
Cai et al [56]	To investigate whether IVF independently increase the risk of GDM and its association to maternal body mass index	• Cross-sectional study<br>• Hospital<br>• N=1089	• IVF pregnancies had nearly double the odds of GDM (OR = 1.83, 95% CI=1.03–3.26) and elevated fasting (mean difference = 0.12 mmol/L, 95% CI= 0.00–0.24) and OGTT 2-h blood glucose levels (mean difference = 0.64 mmol/L, 95% CI= 0.27–1.01).<br>• After stratification by first-trimester BMI, these increased risks of GDM (OR = 3.54, 95% CI: 1.44–8.72) and elevated fasting (mean difference = 0.39 mmol/L, 95% CI: 0.13–0.65) and 2-h blood (mean difference = 1.24 mmol/L, 95% CI: 0.56–1.91) glucose levels were significant only in the IVF group who is also overweight or obese.
Cai et al [69]	To examine the influence of maternal sleep quality and nocturnal sleep duration on risk of GDM.	• Retrospective cohort<br>• Hospital<br>• N=686; n=131 (GDM) & n=555 (control)	• Poor sleep quality (OR=1.75, 95% CI=1.11-1.75) and short nocturnal sleep duration (OR=1.96, 95% CI -1.05-3.66) were independently associated with increased risk of GDM.
Pang et al [69]	To examine the cross-sectional associations of dietary protein intake from different food sources during pregnancy with the risk of GDM in a multiethnic Asian population.	• Prospective cohort<br>• Hospital<br>• N=980; n=175 (GDM) & n=805 (control)	• A higher total dietary protein intake was associated with a higher risk of GDM (OR 2.15, 95% CI=1.27-3.62; p = 0.016). Higher intake levels of both animal protein (OR= 2.87, 95% CI= 1.58-5.20, p= 0.001) and vegetable protein (OR=1.78, 95% CI=0.99- 3.20, p= 0.009) were associated with a higher risk of GDM.

			• Among the animal protein sources, higher intake levels of seafood protein (OR=2.17, 95%CI=1.26-3.72, p = 0.023) and dairy protein (OR= 1.87,95% CI=1.11-3.15, p= 0.017) were significantly associated with a higher GDM risk.
Chawanpaiboon et al [49]	To study the adverse outcome in pregnant women age 16 years or younger.	• Prospective cohort<br>• Hospital<br>• N=1810	• Hypertension (p<0.031), pulmonary diseases (p<0.017), and gestational diabetes (p<0.001) were significantly low in the young maternal age group.
Jamalpour et al [38]	To investigate glucokinase regulatory gene rs780094 is a risk factor for GDM	• Case-control<br>• Hospital<br>• N=1,122; n=267 (GDM) & n=855 (control)	• Frequency of risk allele C was significantly higher in GDM (OR=1.34, 95% CI=1.09-1.66, p=0.006).<br>• The C allele associated with increased level of random 2-hour fasting plasma glucose and pregravid BMI.
Lai et al [61]	To examine the cross-sectional associations of plasma folate, vitamins B6, B12, and homocysteine concentrations with GDM and glycaemia	• Prospective study<br>• Hospital<br>• N=913	• Higher plasma folate was associated with higher 2-hour glucose and higher odds of GDM (OR=0.15, 95% CI=0.02- 0.23) per 1-SD increment in folate, (OR=1.29, 95% CI=1.00, 1.60)], mainly among Indian mothers.<br>• Higher plasma vitamin B12 and homocysteine were associated with lower fasting and 2hour glucose, and lower odds of GDM (β= -0.04, 95% CI=-0.07- -0.01) per 1-SD increment in B12 and (β =-0.09, 955 CI= -0.18--0.003) respectively, (OR= 0.81, 95% CI=0.68-0.97); (OR=-0.05, 95% CI= -0.08- -0.02) per 1-SD increment in homocysteine and (β = 0.12, 95% CI=-0.21- -0.02) respectively, (OR =0.76, 95% CI=0.62, 0.92).<br>• The highest odds of GDM were observed among women with combined vitamin B12 insufficiency and high folate concentration (OR= 1.97, 95% CI= 1.05-3.68).
Rueangdetnarong et al [41]	To compare the levels of oxidative stress biomarkers between pregnancies with GDM and normoglycemic pregnancies.	• Prospective cohort<br>• Hospital<br>• N=62; n=30 (GDM) & n=32 (control)	• Maternal serum 8Isop and TNF-α levels were significantly higher in GDM group (OR=737.5, 95% CI=584.9-1811.5, p= 0.032) and (OR=4.70, 95% CI=2.42-6.91, p=0.047), despite good glycemic control.<br>• At early labor, maternal 8Isop levels were significantly higher in GDM (OR=666.4, 95% CI=454.5-1528.8, p=0.001).

			• The biomarkers in the cord blood as well as maternal and neonatal outcomes in both groups were not significantly different.
Somprasit et al [55]	To evaluate the effects of high pre-pregnancy BMI on the risk of poor obstetric outcomes among Asian women using BMI criteria by Regional Office for the Western Pacific Region of WHO (WPRO).	• Retrospective cohort<br>• Hospital<br>• N=2735	• High pre-pregnancy BMI pregnant women have significantly higher adjusted risk ratio for GDM (RR= 1.54, 95% CI= 1.30-1.84) and preeclampsia (RR= 1.17, 95% CI= 1.12-1.23), induction of labor (RR= 1.41, 95% CI= 1.041.90), prolong second stage of labor, including, caesarean delivery (RR= 1.28, 95% CI =1.11-1.48) or obstetrics procedures and (RR= 1.17, 95% CI =1.05-1.27).<br>• In addition, the adjusted risk ratio of postpartum hemorrhage (RR= 1.86, 95% CI= 1.01-3.43) and neonatal macrosomia were significantly increased (RR= 1.46, 95% CI= 1.28-1.65).
Yong et al [57]	To determine the association between dietary patterns before and during pregnancy and risk of GDM in Malaysian pregnant women.	• Prospective cohort<br>• Hospital<br>• N=452; n=267 (GDM) & n=855 (control)	• Women with high adherence (HA) to dietary pattern during pre-pregnancy (OR=0.45, 95% CI=0.20–0.91) and dietary pattern during first trimester (OR=0.28, 95% CI=0.11–0.68) showed a significantly reduced risk of GDM compared to women with low adherence (LA).

Table S4: Studies reported complication for GDM in Southeast Asia (n=24)

Author	Study aim	Study design / Setting / Sample size	Main findings
Sia et al [76]	To determine the incidence of postpartum diabetes and/or glucose intolerance among and to compare the risk factors present among Filipino GDM women.	• Prospective cohort<br>• Hospital<br>• N=107; n= 56 (GDM) & n=51 (control)	• Multigravid patients (OR=2.84; 95% CI 1.20,6.70) and those with postpartum obesity (OR=2.84; 95% CI= 1.20-6.70) are more likely to have prediabetes.<br>• Diagnosis of GDM at an earlier trimester increases the odds of having postpartum diabetes (OR=3.05; 95% CI= 1.02-9.18).<br>• Body mass index falls under obese class II postpartum, the probability having postpartum diabetes increases 115 times (95% CI= 3.96-3357.83; p=0.006).
Chew et al [80]	To determines the prevalence of prediabetes and type 2 diabetes mellitus (T2DM), and the associated antenatal and historical risk factors among women with previous GDM.	• Cross sectional retrospective study<br>• Hospital<br>• N=448	• Fasting plasma glucose at diagnosis of index GDM (β=0.065, p=0.001), (β=0.734, p=0.007), (β=0.689, p=0.007) and duration lapse after index GDM (β =0.009; p=0.015), (β =0.011; p=0.025), (β =0.012; p=0.004) were shown to be significantly higher in women with isolated impaired fasting glucose (IFG), combined IFG/impaired glucose tolerance and T2DM, respectively as compared to women with normal glucose tolerance.<br>• 2-hour plasma glucose at diagnosis of index GDM was significantly higher only in women who progressed to T2DM (β =0.515; p=0.002) when compared to those that remained normal glucose tolerant.
Youngwanichsetha et al [72]	To determine the association between hypoglycaemia among neonates born to mothers with gestational diabetes mellitus and their postpartum prediabetes.	• Prospective cohort<br>• Hospital<br>• N=118 (GDM)	• The incidence of neonatal hypoglycaemia was 42.37% and (OR=0.30; 95% CI= 014-0.66).<br>Significant association between neonatal hypoglycemia and postpartum blood sugar levels (β=0.101, 95% CI = 0.044–5.284) of women with a history of gestational diabetes mellitus.
Youngwanichsetha et al [84]	To investigate the factors related to exclusive breastfeeding among postpartum Thai women with a history of GDM.	• Retrospective cross-sectional study<br>• Hospital<br>• N=150	• Significant factors related to the 6-month exclusive breastfeeding were maternal age, employment, parity, body mass index, duration of newborn's admission in NICU and exclusive breastfeeding intention (p<0.05).

Youngwanichsetha et al [78]	To investigate the factors related to prediabetes among Thai women with a history of GDM.	• Retrospective cross-sectional study<br>• Hospital<br>• N=210	• Factors associated with prediabetes were: (i) being over 35 years of age (p=0.043); (ii) three or more pregnancies (p=0.004); (iii) recurrent GDM (p=0.002); (iv) high plasma glucose before taking a 100 g glucose tolerance test and high postprandial plasma glucose during pregnancy (p=0.005); and (v) being overweight or obese at six weeks' postpartum (p=0.020).
Aris et al [68]	To examine the relationship between gestational glycemia and neonatal adiposity in a multiethnic cohort of Singaporean neonates.	• Prospective cohort<br>• Hospital<br>• N=1081	• Each 1 SD increase in fasting glucose was associated with increases in large for gestational age (OR=1.31, 95% CI= 1.10– 1.55), and % body fat (OR=1.72, 95% CI= 1.31–2.27) an∑SFT greater than the 90th centile (OR=1.64, 95% CI= 1.32–2.03).
Asvanarunat et al [65]	To compare pregnancy outcomes between women who gave birth having gestational weight gain (GWG) within and above or below Institute of Medicine (IOM) guidelines.	• Retrospective cohort<br>• Hospital<br>• N=3683	• 34.9% had weight gain within, 36.5% above, and 28.7% below IOM guidelines.<br>• Women with higher gestational weight gain in underweight (OR=1.16, 95% CI=0.69-1.94), normal weight (OR=1.30, 95% CI=1.03-1.65) and overweight (OR=1.64, 95% CI=1.05-2.58) had an increased risk of cesarean birth<br>• Women with higher gestational weight gain in underweight (OR=4.90, 95% CI=0.49-49.36), normal weight (OR=3.31, 95% CI=1.75-6.28), overweight (OR=1.99, 95% CI=0.87-4.58) had an increased risk macrosomia except for obese group.<br>• Women with higher gestational weight gain in underweight (OR=1.52, 95% CI= 0.40-5.80) normal weight (OR=2.28, 95% CI=1.31-3.97), overweight (OR=1.09, 95% CI=0.52-2.29) and obese (OR=1.99, 95% CI=0.59-6.72) had an increase of large gestational age (LBW).<br>• Women with higher gestational weight gain in underweight (OR=0.81, 95% CI= 0.40-1.61), normal weight (OR=0.52, 95% CI=0.35-0.77), overweight (OR=0.77, 95% CI=0.37-1.58) and obese (OR=0.56, 95% CI=0.17-1.86) had a decreased risk for preterm birth.<br>• Women with higher gestational weight gain in underweight (OR=0.40, 95% CI= 0.15-1.09), normal weight (OR=0.41, 95% CI=0.26-0.66) and obese

			(OR=0.45, 95% CI=0.11-1.76) had a decreased risk of large birth weight except for overweight group.<br>• Women with higher gestational weight gain in underweight (OR=0.72, 95% CI= 0.40-1.30), normal weight (OR=0.78, 95% CI=0.58-1.05) overweight (OR=0.84, 95% CI=0.39-1.79) and obese (OR=1.68, 95% CI=0.42-6.67) had a decreased risk for small gestational age (SGA).<br>• Women with lower gestational weight gain in underweight (OR=1.74, 95% CI= 1.03-2.94), normal weight (OR=1.54, 95% CI=1.11-2.15) overweight (OR=0.94, 95% CI=0.34-2.57) and obese (OR=2.12, 95% CI=0.45-10.00) had an increased risk for preterm birth.<br>• Women with lower gestational weight gain in underweight (OR=0.44, 95% CI= 0.08-2.31), normal weight (OR=0.45, 95% CI=0.21-0.98), overweight (OR=0.35, 95% CI=0.07-1.59) and obese (OR=0.93, 95% CI=0.09-9.26) had an increased risk for LGA.<br>• Women with lower gestational weight gain in underweight (OR=2.16, 95% CI= 1.39-3.33), normal weight (OR=1.76, 95% CI=1.35-2.30), overweight (OR=1.16, 95% CI=0.41-3.26) and obese (OR=9.38, 95% CI=1.79-48.96) had an increased risk for SGA.<br>• Women with lower gestational weight gain in underweight (OR=0.55, 95% CI=0.34-0.91), normal weight (OR=0.80, 95% CI=0.62-1.03), overweight (OR=0.92, 95% CI=0.47-1.78) and obese (OR=0.53, 95% CI=0.12-2.33) had a decreased risk for cesarean birth.<br>• Neonates delivered from women whose gestational weight gains were above IOM guidelines were also heavier than those from neonates whose maternal weight gains during pregnancy were within IOM guidelines.
Yadav et al [69]	To identify the risk factors influencing the development of macrosomia among pregnant women and to develop a regression model to predict macrosomia.	• Retrospective cross-sectional study<br>• Hospital<br>• N=2332	• Occurrence of macrosomia was significantly observed in maternal age 35 years (OR=1.79, 95% CI = 1.170– 2.738), in women with a weight gain of 10 kg and above (OR=1.6, 95% CI = 1.162–2.202), in multiparous women (OR=2.15, 95% CI = 1.251– 3.69), in Chinese women (OR = 1.86, CI = 1.228–2.819), in GDM mother (OR=6.75, 95% CI=4.369–8.472), gestational weeks of more than 40 weeks (OR=0.01, 95% CI = 1.311–3.08), fathers with BMI of more than 25 (OR=1.89, 95% CI = 1.198–2.985).

Aris et al [67]	To examine the relation between gestational glycemia and prepregnancy body mass index (ppBMI) with offspring growth in an Asian mother-offspring cohort.	• Prospective cohort<br>• Hospital<br>• N=937	• Gestational fasting plasma glucose (FPG) was positively associated with birth weight (β=0.17; 95% CI=0.10-0.24, p=0.001) and birth BMI (β=0.15, 95% CI= 0.06-0.40,p= 0.001) but not at ≥3 months of age.<br>• Maternal ppBMI was positively associated with birth variables and conditional growth in weight and BMI in the first 36 months of life.<br>• In nonobese mothers, each unit increase in gestational FPG was associated with increased offspring weight (β=0.08, 95% CI= 0.008-0.16; P = 0.03) and BMI (β=0.08; 95% CI=0.003-0.15,p = 0.04) as well as increased risk of overweight in the first 36 months of life (OR= 1.36; 95% CI= 1.10, 1.68).<br>• In obese mothers, each unit increase in gestational FPG was associated with decreased offspring weight (β=20.01, 95% CI, 20.02- 20.003, p=0.01) and BMI (β=20.008, 95% CI= 20.01- 20.002, p=0.01) and decreased risk of overweight (OR= 0.59; 95% CI= 0.41- 0.86) in the first 36 months of life.
Hanprasertpong et al [74]	To determine the pregnancy outcomes and identify predictive factors of its adverse outcomes.	• Retrospective cohort<br>• Hospital<br>• N=240	• Pre-eclampsia, gestational diabetes mellitus, pre-term birth and foetal intrauterine growth restriction found were 15, 7.9, 13.7 and 3.7%, respectively.<br>• Antenatal care place (OR= 7.18, 95% CI= 1.79-28.80; p = 0.005), low hemoglobin level (OR= 7.18, 95% CI 1.79-28.80; p = 0.005) and the presence of maternal underlying disease (OR= 7.18, 95% CI= 1.79-28.80; p = 0.005) were significantly related to increased risk of overall adverse maternal outcomes.
Srichumchit et al [66]	To compare pregnancy outcomes between women with GDM and those with low-risk pregnancies during implementation of the GDM practice guideline.	• Retrospective cohort<br>• Hospital<br>• N=21,771 n=1350 (GDM) & n= 20,421 (control)	• The incidence of fetal macrosomia-the main outcome-was significantly higher (OR= 1.48, 95% CI= 1.28=, 1.71, p<0.001) in the GDM group than in the control group.<br>• The prevalence of pregnancy induced hypertension in the GDM group was twice as high as that in the control group (OR= 1.67, 95% CI= 1.39-, 2.00; p< 0.001).<br>• The incidences of cesarean delivery (OR= 1.36, 95% CI= 1.20-, 1.54, p<0.001), cephalopelvic disproportion delivery (OR= 1.56, 95% CI= 1.31-, 1.86, p<0.001), pregnancy-induced hypertension (OR= 1.67, 95% CI= 1.39-, 2.00, p<0.001) and

			shoulder dystocia (OR= 7.84, 95% CI= 3.29-, 18.70, p<0.001) were also significantly higher in the GDM group.
Ruksasakul et al [79]	To compare the prevalence and risk factors of metabolic syndrome after delivery in GDM and normal pregnant.	• Case control<br>• Hospital<br>• N=107; n= 56 (GDM) & n=51 (control)	• The mean current age, median body mass index (BMI) before pregnant, current BMI, waist/height ratio and systolic blood pressure were significantly higher in GDM group.<br>• Metabolic syndrome was higher in the GDM group (26.8% (15/56) vs. 7.8% (4/51), (OR= 4.3, 95% CI=: 1.32-13.99).<br>• Only a BMI >/=25 kg/m2 before index pregnancy was a significant independent factor for this condition (OR= 7.18, 95% CI= 1.79-28.80; p = 0.005).<br>• After delivery, GDM group had more insulin resistance, assessed by HOMA-IR, less insulin sensitivity assessed by Masuda index and QUICKI score and less insulin secretion assessed by HOMA-B, comparing to normal control group.
Li et al [82]	To determine relationship between GDM and retinal vascular changes in expectant mothers, a proxy for small-vessel dysfunction, at 26–28 weeks of pregnancy	• Prospective cohort<br>• Hospital<br>• N=1136	• Mothers with GDM had narrower arteriolar caliber (OR= -1.6, 95% CI= -3.1, -0.2), reduced arteriolar fractal dimension (OR= -0.01, 95% CI= -0.02- -0.001), and larger arteriolar branching angle (OR=1.8, 95% CI=0.3-3.3 degrees) than mothers without GDM.<br>• After further adjusting for traditional risks of GDM, arteriolar branching angle remained significantly larger in mothers with GDM than those without GDM (OR=2.0 , 95% CI=0.5- 3.6).
Wanthong et al [81]	To determine the prevalence of and risk factors for abnormal glucose tolerance (AGT) in previous gestational diabetes mellitus (pGDM) women.	• Prospective study<br>• Hospital<br>• N=100	• Plasma glucose (PG) at 1 h after a 50g-glucose challenge test (GCT), PG at 1 h after 100g-OGTT, HbA1c, and HOMA-IR were significantly greater in women with abnormal glucose tolerance (AGT) than normal glucose tolerance (NGT) women.<br>• The proportion of women with ≥3 abnormal PG values during 100g-OGTT was greater in AGT than NGT group (50.7% vs. 15.8%).<br>• PG ≥150 mg/dl at 1 h after a 50g-GCT (OR=22.02. 95% CI-3.78-128.31) and ≥3 abnormal PG values in 100g-OGTTs (OR=4.75, 95% CI=1.08-20.96) were risk factors for developing AGT.

Li et al [75]	To assess the associations of GDM and hypertensive disorders of pregnancy (HDP)'s individual and synergic contribution to risks of postpartum cardio-metabolic diseases (metabolic syndrome (MetS), abnormal glucose metabolism and hypertension (HTN).	• Prospective cohort<br>• Hospital<br>• N=276	• Found associations of GDM episodes with postpartum abnormal glucose metabolism (single episode: (RR=2.9, 95% CI=1.7-4.8); recurrent episodes (≥2): RR = 3.8, 95% CI=2.1–6.8).<br>• Found association between histories of HDP and HTN (RR = 3.6, 95% CI=1.5-8.6).<br>• Either (RR= 2.6, 95% CI=1.7–3.9) or both gestational complications (RR=2.7, 95% CI= 1.6–4.9) was associated with similar risk of postpartum cardiometabolic disease.
Sunjaya et al [71]	To compare the clinical and glycemic profile as well as pregnancy complications and infant mortality among GDM women.	• Retrospective cross-sectional study<br>• Hospital<br>• N=45 (GDM)	• No maternal mortalities were reported, only 6 pregnancies were complicated with infant death.<br>• Comorbidities mainly found were preeclampsia, anemia and urinary tract infection.<br>• Most patients delivered through caesarian section.<br>• Those with poor outcomes have a significantly higher body mass index prior to pregnancy (p<0.05), higher body weight prior and after pregnancy (p,0.05) as well as worse glycemic profile (p<0.05)<br>• Significant poor outcome with OADs usage (p<0.05).
Fatin et al [73]	To determine the outcomes of six weeks postpartum glucose testing and its associated factors among women with a history GDM.	• Prospective study<br>• Hospital<br>• N= 122	• Insulin usage (OR=5.44, 95% CI=1.53- 19.43; p=0.009), abnormal glycated hemoglobin (OR=8.70, 95% CI:2.68-26.27, p<0.01), hospital follow-up (OR=3.38, 95% CI= 1.11-10.34, p=0.033) and neonatal intensive care unit admission (OR=3.96, 95% CI= 1.16-13.54, p=0.028) were found to have significant associations with abnormal glucose tolerance at six weeks postpartum.<br>•
Jirakittidul et al [85]	To determine the prevalence of breastfeeding and the factors associated with breastfeeding during	• Prospective cohort<br>• Hospital<br>• N=229	• Prevalence of any breastfeeding at 24 h, 6 weeks, 3 months, and 6 months postpartum was 28.8% (n = 66), 94.3% (n = 214), 71% (n = 154), and 49.8% (n = 104), respectively.

	the first six months postpartum in women with GDM.		• Maternal intention to breastfeed for 6 months was an independent predictor for both 6 months EBF (RR=16.38; 95% CI=2.29-116.99) and any breastfeeding (RR= 2.65; 95% CI =1.65-4.25).<br>• Breastfeeding initiation within 24 h postpartum (RR= 1.38; 95% CI =1.08-1.76) and being a government officer or private business owner (RR= 1.66; 95% CI= 1.03-2.68) were independent predictors of any breastfeeding and EBF for 6 months, respectively.
Lee et al [90]	To determine the prevalence and factors associated with antenatal depressive, anxiety and stress symptoms among women with GDM.	• Cross sectional<br>• Hospital<br>• N=526 (GDM)	• Prevalence of anxiety symptoms was highest (39.9%), followed by depressive symptoms (12.5%) and stress symptoms (10.6%) among women with GDM.<br>• Younger age (OR= 0.955, 95% CI= 0.919-, 0.993), comorbidity with asthma (OR 2.436, 95% CI 1.219, 4.870) and a family history of depression and anxiety (OR= 4.782, 95% CI= 1.281-17.853) had significant associations with antenatal anxiety symptoms.<br>• Being non-Muslim (OR= 2.937, 95% CI= 1.434-, 6.018) and having a family history of depression and anxiety (OR= 4.706, 95% CI= 1.362-, 16.254) had significant associations with antenatal depressive<br>• Non-Muslim (OR= 2.451, 95% CI= 1.273-, 4.718) had a significant association with antenatal stress symptoms.
Lee et al [91]	To determine the association of candidate genes and psychological symptoms of depression, anxiety, and stress among women with GDM followed by the determination of their odds of getting psychological symptoms, adjusted for socio-demographical background, maternal, and clinical characteristics.	• Prospective cohort<br>• Hospital<br>• N=343	• Single nucleotide polymorphisms (SNPs) recorded a significant association between SNP of EPHX2 (rs17466684) and depression symptoms (AOR = 7.854, 95% CI = 1.330–46.360) and stress symptoms (AOR = 7.664, 95% CI = 1.579–37.197).<br>• Associations were also observed between stress symptoms and SNP of OXTR (rs53576) and (AOR = 2.981, 95% CI = 1.058–8.402) and SNP of NRG1 (rs2919375) (AOR = 9.894, 95% CI = 1.159–84.427).<br>• The SNP of EPHX2 (rs17466684) gene polymorphism is associated with depression symptoms among Malaysian women with GDM.<br>• SNP of EPHX2 (rs17466684), OXTR (rs53576) and NRG1 (rs2919375) are also associated with stress symptoms.

Nguyen et al [86]	To investigate the relationship between GDM and the duration for which Vietnamese women breastfeed their babies postpartum.	• Prospective cohort<br>• Hospital<br>• N=1709; n=373 (GDM) & n= 1336 (control)	• The risk of early breastfeeding cessation was higher in GDM women than their non-GDM counterparts after adjustment for demographic factors (hazard ratios [HR]=1.39, 95% CI=1.13-1.71, p=0.002), and all potential confounding factors (HR = 1.38, 95% CI=1.12-1.70, p=0.002).<br>• There were no significant differences in breastfeeding outcomes at discharge (early initiation, prelacteal feeding, and "any" breastfeeding rate) between GDM and non-GDM mothers.
Lee et al [70]	To determine the prevalence of neonatal outcomes and its association among mothers with gestational diabetes mellitus with and without the presence of depression, anxiety, and stress symptoms in Malaysia.	• Prospective cohort<br>• Hospital<br>• N=418	• Positive association found from the neonatal respiratory distress with the predictors of presence of depression symptoms in mothers with GDM (AOR=3.87, 95% CI=1.32-11.35), living without a husband (AOR=9.74, 95% CI=2.04-46.51), preterm delivery (AOR=7.20, 95% CI=2.23-23.30), caesarean section (AOR=3.33, 95% CI=1.09-10.15), being nulliparous and primiparous (AOR=3.62, 95% CI=1.17-11.17) and having family history of diabetes (AOR=3.20, 95% CI=1.11-9.21).
Tengku et al [83]	To evaluate mean macular and retinal nerve fibre layer (RNFL) thickness in pregnant women with GDM and to analyze the association of age, HbA1c level, duration of GDM, type of treatment, family history, previous history of GDM and spherical equivalent with the macular and RNFL thickness.	• Prospective cohort<br>• Hospital<br>• N=220; n= 78 (GDM), n=72 (pregnant control) & n=70 (non-pregnant control)	• There was no significant difference in the mean macular and RNFL thickness in pregnant women with GDM when compared to the control groups (p>0.05).<br>• Age (OR=0.46, 95% CI=−0.62- 0.71), HbA1c (OR=−0.07, 95% CI=−6.84- 6.70), duration of diabetes (OR=−0.25, 95% CI=−0.84- 0.33), treatment received (OR=0.53, 95% CI=−4.83- 5.88), history of GDM (OR=1.97, 95% CI=−0.80- 4.75) and spherical equivalent (OR=0.497, 95% CI= −1.38-2.38) did not show significant association with mean macular and retinal thickness (p>0.05).

Table S5: Studies that reported management for GDM in Southeast Asia (n=17)

Author	Study aim	Study design / Setting / Sample size	Main findings
Hirst et al [96]	To determine attitudes and health behaviors of pregnant women with GDM in Vietnam.	• Prospective study<br>• Hospital<br>• N=35	• Women felt confusion, anxiety and guilt about GDM.<br>• Many perceived their baby to be at increased risk of death.<br>• Advice to reduce dietary starch was confusing.<br>• Women reported being 'hungry' or 'starving' most of the time, unaware of appropriate food substitutions.<br>• Women were concerned about transmission of GDM through breast milk.<br>• Several women planned not to breastfeed and felt they needed more information.<br>• Current sources of information included friends, magazines, a health phone line or the Internet.<br>Women felt small group sessions and information leaflets could benefit them.
Sangeetha et al [98]	To examine the feasibility of lowering GI through GI-based-education among Asian post-GDM women.	• Prospective study<br>• Hospital<br>• N=48	• Low-GI Group (LGIE) group had significant reductions in energy intake (241.7±522.4Kcal, p=0.037, ES=0.463), total carbohydrate (48.7±83.5g, P=0.010, ES=0.583), GI (3.9±7.1, p=0.017, ES=0.549) and GL (39.0±55.3, p=0.003, ES=0.705) and significant increases in protein (3.7±5.4g, 0.003, ES=0.685) and diet fibre (4.6±7.3g, p=0.06). The Conventional Healthy Dietary Recommendation Group (CHDR) group had a significant reduction in fat only (5.7±9.4g, P=0.006, ES=0.606).<br>• There was a 30% increase in GI-concept scores in the LGIE group (p< 0.001).<br>• Changes in GI-concept scores correlated significantly to the reduction in dietary GI (r = -0.642, p=0.045)
Farhanah et al [100]	To examine the current dietetic practices in the management of GDM and to compare nutrient recommendations provided by international guidelines.	• Qualitative study<br>• Hospital<br>• N=148	• Flexible carbohydrate exchanges (82%) was the most common recommendation on carbohydrate intake followed by advises regarding small frequent meals spread over the day (62%) and portion control using the plate method (54%).<br>• Only 11% dietitians incorporated the use of the glycemic index as a measure of carbohydrate intake into their intervention.

Hussain et al [95]	To evaluate the knowledge, attitude, and treatment satisfaction of GDM patients toward their disease.	• Prospective study<br>• Hospital<br>• N=30	• 23 patients (76.6%) had adequate knowledge.<br>• Only, 7 (23.3%) patients had inadequate knowledge.<br>• For attitude, 23 (76.66%) of patients had a negative attitude toward disease and only 7 (23.3%) had a positive attitude.<br>• In terms of satisfaction, 25 (83.33%) patients were satisfied with the given treatment and 5 (16.66%) were unsatisfied.
Youngwanichsetha et al [94]	To evaluate attitude and treatment satisfaction of women suffering from GDM and their association with glycaemic level.	• Prospective study<br>• Hospital<br>• N=30	• 23 patients (76.6%) had adequate knowledge.<br>• Only, 7 (23.3%) patients had inadequate knowledge.<br>• For attitude, 23 (76.66%) of patients had a negative attitude toward disease and only 7 (23.3%) had a positive attitude.<br>• In terms of satisfaction, 25 (83.33%) patients were satisfied with the given treatment and 5 (16.66%) were unsatisfied.
Hussain et al [92]	To evaluate the knowledge about GDM and its corresponding relation with glycaemic level in GDM patients.	• Cross-sectional<br>• Clinic<br>• N=166 (GDM)	• Knowledge had a significant negative association with fasting plasma glucose ($r=-0.306$, $p<0.01$).<br>• Highest mean score was seen for diet/food values domain and lowest for management of GDM.<br>• Educational level seems to be the most significant predictor of GDM knowledge and glycemic control.<br>• Highest mean knowledge score and lowest mean glycemic levels were recorded for patients aged 25-29 years, Malay ethnicity, working women and family history of DM.
Hussain et al [93]	To evaluate attitude and treatment satisfaction of women suffering from GDM and their association with glycaemic level.	• Prospective study<br>• Hospital<br>• N=166 (GDM)	• Only 35 (21.1%) patients had positive attitude and 122 (73.5%) of patients had adequate treatment satisfaction.<br>• No significant association of total mean ATT-19 score with age, ethnicity, educational level, occupational status, family history and type of therapy.<br>• Total mean treatment satisfaction score and educational level shows significant.<br>• Patients with negative attitude and inadequate treatment satisfaction had higher mean glycemic level.

Panpitpat et al [103]	To compare maternal and neonatal complications of GDM between conservative (CMG) and systematic management (SMG).	• Retrospective cohort<br>• Hospital<br>N=205 (GDM)	• Oral glucose tolerance tests (50 and 100 gram) were similar in both groups.<br>• The prevalence of GDM was 57.5 and 55.1% in CMG and SMG, respectively.<br>• Mean gestational age at DM clinic consultation and number of hospital admission of SMG was less than CMG (p< 0.001).<br>• Neonatal hypoglycemic episode in SMG was less than CMG (p=0.007).<br>• Postpartum 75-gram glucose tolerance test appointments and percentages of underwent in SMG were more than CMG (p< 0.001).
Padmapriya et al [104]	To investigate physical activity (PA) and sedentary behavior (SB) in relation to fasting (FG) and 2-h postprandial plasma glucose (2hPG) levels and GDM.	• Prospective cohort<br>• Hospital<br>• N=1083; n=201 (GDM) & n=882 (control)	• SB was not associated with FG, 2hPG and GDM.<br>• Higher categories of PA were associated with lower 2hPG and a lower likelihood of GDM (p-trend < 0.05), but not with FG levels.<br>• Highly active women had lower 2hPG levels (OR= -0.32, 95% CI= −0. 59, −0.05), p = 0.020) and were less likely to have GDM (OR=0.56, 95% CI=0.32-0.98, p=0.040)<br>• Stratified analysis revealed no associations among under/normal-weight women, but significant associations among overweight/obese women; in those with BMI ≥23 kg/m2 (OR=0.52, 95% CI=0.29-0.93, p=0.010), sufficiently active and highly active women were less likely to have GDM (OR=0.34, 95% CI=0.15-0.77, p=0.010).
Youngwanichsetha et al [101]	To explore and describe lived experience of blood glucose self-monitoring among GDM pregnant women.	• Descriptive phenomenological study<br>• N=30 (GDM)	• The findings revealed three themes: being worried about diabetes and blood testing, trying to control it and being patient for the child.<br>• Worry comprised three dimensions: (1) wondering about the impacts of diabetes on the child, (2) concern about maternal health and (3) being worried about doing blood test.<br>• Trying to control diabetes was composed of three dimensions: (1) learning to test blood glucose, (2) being afraid of elevated blood sugar and (3) being aware of what to eat.<br>• Being patient for the child was composed of three dimensions: (1) overcoming food desires, (2) tolerating the fingerprick pain and (3) satisfaction with the outcomes.

Arasoo et al [105]	To understand the perceived barriers to exercise in women with GDM.	• Prospective cohort<br>• Hospital<br>• N=89	• 80.9% were aware that exercise was necessary for women with GDM.<br>• Only 6.7% say that healthcare professionals were their source of information on exercise in pregnancy.<br>• 77.3% of the women with low physical activity had full time jobs.<br>• Housewives (64.5%) had the highest level of physical activity ($p<0.05$).<br>• Tiredness (43.8%), childcare duties (38.2%) and lack of time (27.0%) were the most common perceived barriers to exercise ($p<0.05$).<br>• Nulliparity was significantly associated with tiredness ($p<0.05$).
De Luna [107]	To evaluate the efficacy, safety, and pregnancy outcomes of insulin analog versus human insulin in women with GDM.	• Retrospective cohort<br>• Hospital<br>• N=144	• Good glycemic control and low rate of hypoglycemia in human insulin were comparable to diet-controlled, insulin analog or combination to insulin analog and human insulin groups.<br>• Maternal outcomes (hypertensive disorders of pregnancy and primary cesarean section) in human insulin usage group were not increased and similar compared to other groups.<br>• Neonatal outcomes (birth weight, large for gestational age, neonatal hypoglycemia, neonatal jaundice, and acute respiratory distress syndrome) in human insulin usage group were also not increased and comparable to other groups.<br>• Rates of prematurity were higher in diet-controlled, insulin analog or combination to insulin analog and human insulin groups.
Hewage et al [97]	To evaluate health care providers' perceptions of care responsibilities and resources related to reducing type 2 diabetes risk among women with previous GDM.	• Prospective study<br>• Clinic<br>• N=32	• Health care providers felt that they had less understanding of compliance with long-term maintenance of lifestyle change, exacerbated further by fragmentation of follow-up care.

Nguyen et al [106]	To assess the association between physical activity (PA) during pregnancy and the prevalence GDM accounting for sitting time.	• Prospective cohort<br>• Cohort data<br>• N=1987; n=432 (GDM) & n=1555 (control	• Women undertaking the highest level of PA during pregnancy appeared to have a lower risk of GDM (OR=0.70, 95% CI=0.53- 0.94, p=0.017) when compared to those at the lowest level of PA.<br>• Women with increased levels of moderate-intensive activity (OR= 0.66, 95% CI= 0.50–0.86, p= 0.002) and household/caregiving activity during pregnancy (OR=0.72, 95% CI=0.55- 0.95, p=0.020) were associated with reduced risks of GDM and were not attenuated by their sitting time.<br>• No significant associations between sitting time, light-intensity activity, vigorous-intensity activity, occupation, sports/exercise, commuting, or meeting exercise guidelines to GDM risk.
Paramasivam et al [102]	To determine if therapeutic, retrospective continuous glucose monitoring (CGM) improves HbA1c with less hypoglycemia in women with insulin treated GDM.	• Prospective cohort<br>• Hospital<br>• N= 50 (GDM)	• Lower increase in HbA1c from 28 to 37 weeks' gestation in the CGM group [HbA1c : CGM + 1 mmol/mol (0.09%), control + 3mmol/mol (0.30%); P = 0.024].<br>• Mean HbA1c remained unchanged throughout the trial in the CGM group but increased significantly in controls as pregnancy advanced.<br>• Mean HbA1c in the CGM group was lower at 37 weeks compared with controls [33 +/- 4 mmol/mol (5.2 +/- 0.4%) vs. 38 +/- 7 mmol/mol (5.6 +/- 0.6%), P < 0.006].<br>• Some 92% of the CGM group achieved an HbA1c </= 39 mmol/mol (</= 5.8%) at 37 weeks compared with 68% of the control group (P = 0.012).<br>• Neither group experienced severe hypoglycemia.
Kijmanawat et al [108]	To evaluate the effect of probiotic supplements on insulin resistance in pregnant women with diet controlled GDM.	• Prospective study<br>• Hospital<br>• N= 57	• The changes in metabolic parameters after randomization showed significant improvement in glucose metabolism in the probiotic group compared with the placebo group, including fasting plasma glucose (β=-3.94, 95% CI= -7.62- -0.27, p = 0.034), fasting plasma insulin (β= -2.67, 95% CI= -3.57, -1.76, p = 0.001) and homeostatic model assessment for insulin resistance (β= -0.63, 95% CI= -0.86- -0.41, p = 0.001).<br>• Weight gain during randomization was similar between the two groups.

Suhaimi et al [99]	To compare carbohydrates consumed women who did and did not have a history of GDM.	• Prospective study<br>• Community<br>• N= 80; n=40 (GDM), n=40 (normal)	• Daily carbohydrate intake among women with no history of GDM was higher than women who did have a history of GDM ($p = 0.02$, $p<0.05$).<br>• No significant difference was seen for dietary GI ($p = 0.24$, $p>0.05$) or dietary glycemic load ($p = 0.09$, $p>0.05$) between the two groups. However, the carbohydrate sources for the two groups differed significantly.<br>• Women without a history of GDM had greater intake of rice varieties with high GI ($p = 0.08$, $p<0.05$), pasta intake ($p = 0.03$, $p<0.05$) and low GI beverages ($p = 0.07$, $p<0.05$) compared to women with a history of GDM.
